# Atypical DNA methylation of genes encoding cysteine-rich peptides in *Arabidopsis thaliana*

**DOI:** 10.1186/1471-2229-12-51

**Published:** 2012-04-19

**Authors:** Wanhui You, Matthew Spencer, Marc W Schmid, Ueli Grossniklaus, Stacey A Simon, Blake C Meyers, Antonius JM Matzke, Marjori Matzke

**Affiliations:** 1 Gregor Mendel Institute of Molecular Plant Biology, Austrian Academy of Sciences, Vienna, Austria; 2 Institute of Plant Biology and Zürich-Basel Plant Science Center, University of Zürich, Zürich, Switzerland; 3 Department of Plant and Soil Sciences, and Delaware Biotechnology Institute, University of Delaware, Newark, USA; 4 Institute of Bioorganic Chemistry, Polish Academy of Sciences, Poznan, Poland; 5 Epigenetics Laboratory, Queensland Institute of Medical Research, Herston, Brisbane, Queensland, Australia

## Abstract

****Background**:**

In plants, transposons and non-protein-coding repeats are epigenetically silenced by CG and non-CG methylation. This pattern of methylation is mediated in part by small RNAs and two specialized RNA polymerases, termed Pol IV and Pol V, in a process called RNA-directed DNA methylation. By contrast, many protein-coding genes transcribed by Pol II contain in their gene bodies exclusively CG methylation that is independent of small RNAs and Pol IV/Pol V activities. It is unclear how the different methylation machineries distinguish between transposons and genes. Here we report on a group of atypical genes that display in their coding region a transposon-like methylation pattern, which is associated with gene silencing in sporophytic tissues.

****Results**:**

We performed a methylation-sensitive amplification polymorphism analysis to search for targets of RNA-directed DNA methylation in *Arabidopsis thaliana* and identified several members of a gene family encoding cysteine-rich peptides (CRPs). In leaves, the *CRP* genes are silent and their coding regions contain dense, transposon-like methylation in CG, CHG and CHH contexts, which depends partly on the Pol IV/Pol V pathway and small RNAs. Methylation in the coding region is reduced, however, in the synergid cells of the female gametophyte, where the *CRP* genes are specifically expressed. Further demonstrating that expressed *CRP* genes lack gene body methylation, a *CRP4-GFP* fusion gene under the control of the constitutive 35 S promoter remains unmethylated in leaves and is transcribed to produce a translatable mRNA. By contrast, a *CRP4-GFP* fusion gene under the control of a *CRP4* promoter fragment acquires CG and non-CG methylation in the *CRP* coding region in leaves similar to the silent endogenous *CRP4* gene.

****Conclusions**:**

Unlike CG methylation in gene bodies, which does not dramatically affect Pol II transcription, combined CG and non-CG methylation in *CRP* coding regions is likely to contribute to gene silencing in leaves because loss of this methylation in synergid cells is associated with *CRP* gene expression. We discuss this unusual methylation pattern and its alteration in synergid cells as well as the possible retrogene origin and evolutionary significance of *CRP* genes that are methylated like transposons.

## **Background**

Plants have evolved a complex transcriptional machinery for generating and using small RNAs that guide DNA cytosine methylation at homologous regions of the genome. Key components of the RNA-directed DNA methylation pathway include two functionally diversified RNA polymerase II (Pol II)-related RNA polymerases called Pol IV and Pol V [1]. Pol IV is needed to produce the small RNA trigger for methylation whereas Pol V acts downstream of small RNA biogenesis to facilitate *de novo* methylation of genomic DNA at the small RNA-targeted site. Additional factors, including chromatin remodelers, putative transcription factors, and several novel, plant-specific proteins whose functions are not well understood, are required for Pol V function [1,2]. In a current model, Pol V synthesizes scaffold transcripts that interact with ARGONAUTE4-bound small RNAs, which recruits the methylation machinery to the target DNA [3].

RNA-directed DNA methylation results in a characteristic modification pattern that is typified by methylation of cytosines in all sequence contexts (CG, CHG and CHH, where H is A, T or C) within the region of small RNA-DNA sequence homology [4]. In particular, asymmetric CHH methylation is a hallmark of RNA-directed DNA methylation. DOMAINS REARRANGED METHYLTRANFERASE2 (DRM2) is the major enzyme catalyzing *de novo* methylation of cytosines in all sequence contexts in response to small RNA signals [5,6]. The maintenance activities of METHYLTRANSFERASE1 (MET1) and CHROMOMETHYLASE3 act primarily to perpetuate pre-existing CG and CHG methylation, respectively, during successive rounds of DNA replication [7].

Transposons, pseudogenes and non-protein coding repeats are frequent targets of RNA-directed DNA methylation [8,9]. By contrast, protein coding genes are generally free of RNA-directed DNA methylation unless intimately associated with repeats or transposon-related sequences [10-12]. However, up to 30 % of expressed genes in *Arabidopsis thaliana* have in their gene bodies exclusively CG methylation that relies on MET1 and is independent of the RNA-directed DNA methylation pathway [8,9]. Thus, transposons and genes can be distinguished by discrete methylation patterns that are imposed by different methylation machineries. The origins of these distinct methylation patterns and their functional significance are not yet fully understood [13-15]. The biological role of CG methylation in gene bodies, which does not inhibit transcriptional elongation by Pol II, is unknown but it may prevent spurious transcription from internal promoters [16] or help to define exons [17]. An alternative proposal is that gene body methylation restrains genes from being responsive to internal or external cues, e.g. developmental or environmental signals [18].

Curiously, even though transposons are frequent targets of RNA-directed DNA methylation, only a small subset of transposons is selectively reactivated in mutants defective in this epigenetic pathway [15,19]. By contrast, a number of transposons are mobilized in mutants defective in MET1 or the chromatin remodeler DECREASE IN DNA METHYLATION1 [13,20]. Therefore, even though RNA-directed DNA methylation contributes to repression of transposons it is not the sole epigenetic modification involved in the silencing of these elements [13,15].

In a search for targets of RNA-directed DNA methylation in *Arabidopsis*, we identified several members of a gene family encoding small cysteine-rich peptides (CRPs). These *CRP* genes are atypically methylated like transposons, containing CG, CHG and CHH methylation in their gene bodies. This gene body methylation, which is due in part to RNA-directed DNA methylation, is associated with gene silencing in leaves and is reduced in synergid cells of the female gametophyte where the *CRP* genes are specifically expressed. Here we report these results and discuss the possible origin and evolutionary significance of this unusual pattern of DNA methylation.

## **Results**

### **Atypical methylation pattern of CRP genes embedded in SAT5 repeats**

To identify targets of RNA-directed DNA methylation in the *Arabidopsis* genome, we used methylation-sensitive amplification polymorphism (MSAP) [21,22]. Cutting genomic DNA with appropriate restriction enzymes that are methylation-sensitive (*Eco*T22I: recognition sequence ATGCAT) and methylation-insensitive (*Mse*I: recognition sequence TTAA) allowed us to identify genomic regions that are differentially methylated at asymmetric CHH sites in wild-type plants compared to an *nrpe1* mutant, which is impaired in the largest subunit of Pol V [23,24]. Two fragments identified in this analysis (Additional file 1: Figure S1) were found in a BLAST search to match unannotated intergenic regions of the *Arabidopsis* genome. A further sequence search against Repbase http://www.girinst.org revealed similarity of these fragments to the AT*SAT5* repeat (referred to hereafter as *SAT5*), which is defined by a 2196-bp long consensus sequence [25]. *SAT5* is annotated as a satellite sequence that has recently expanded near the centromere of chromosome 5 with several distantly related single copies dispersed on other chromosomes [25]. A subsequent BLAST search using the 2196-bp *SAT5* repeat monomer as a query sequence identified four partial or complete single, dispersed copies of *SAT5* in the *Arabidopsis* genome (Table 1; Additional file 2: Figure S2).

**Table 1:** Single dispersed copies of SAT5 repeats

**Chromosome**	**Sequence context**	**Genome coordinates**	**Length (bp)**	***CRP*gene embedded(AGI number)**
2	intergenic	7845025-7844080	946	At2g18042 pseudogene
3	transposon-rich	14681750-14683927	2178	At3g42565 gene
4	transposon and gene	6038150-6037139	1012	At4g09545 gene
5	intergenic	24543172-24542491	682	At5g60978 pseudogene

Four single dispersed copies of the SAT5 repeat are detected in a BLAST search using the 2196 bp SAT5 consensus sequence [25] as a query sequence. One nearly full-length copy is in a transposon-rich region close to the centromere of chromosome 3. Two truncated copies, which correspond to those detected in the MSAP analysis, are present in relatively gene-rich regions on chromosomes 2 and 5, respectively. A third truncated copy is present in a region containing both genes and transposons on chromosome 4. The AGI numbers of the embedded *CRP* genes or pseudogenes are shown.

**Figure 1:**
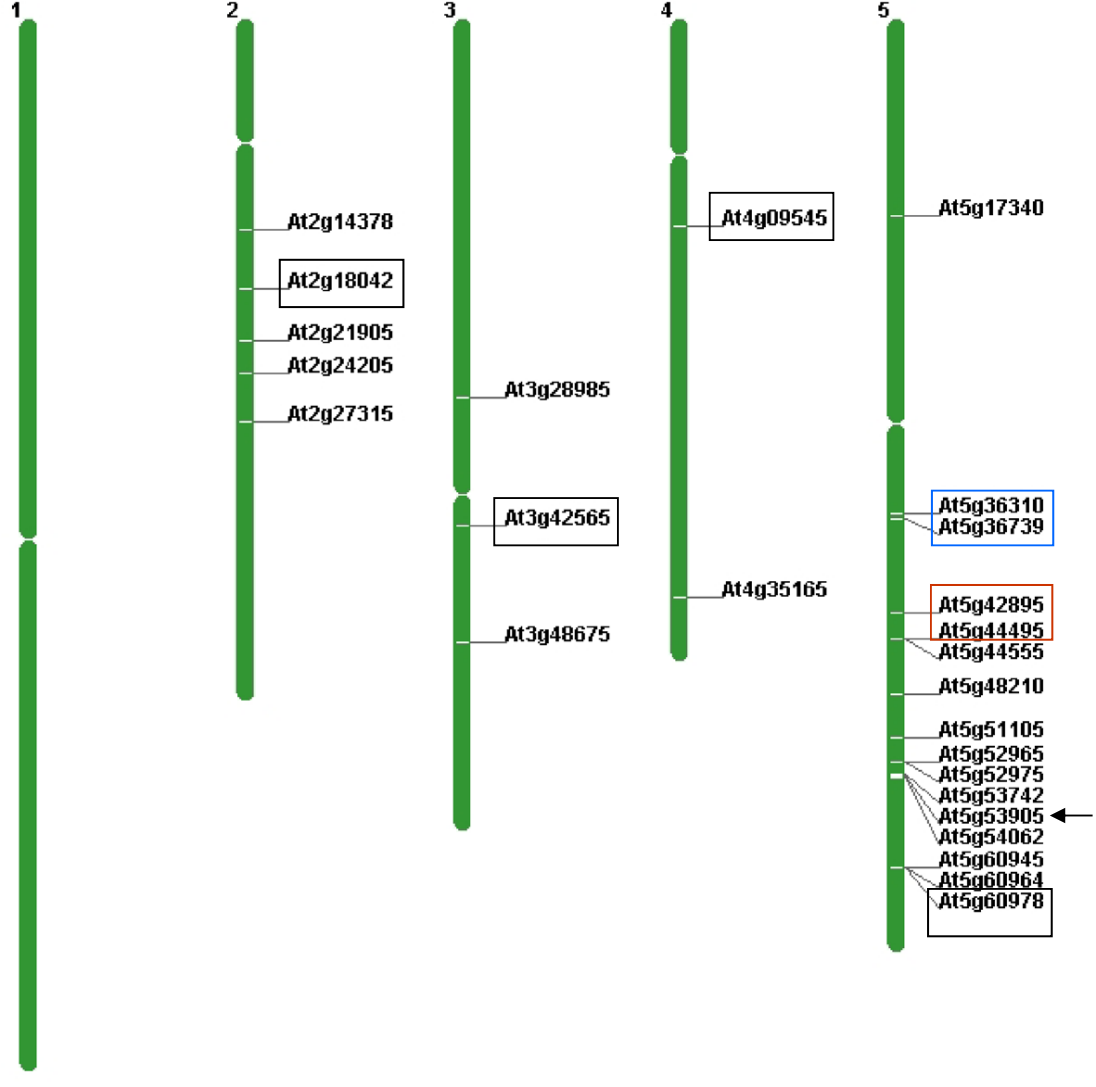
**Chromosomal locations of CRP3600 family members.** The CRP3600 subgroup has 57 members [26], 32 of which are embedded in *SAT5* repeat monomers arranged in a repeat array close to the centromere of chromosome 5; the AGI numbers of only the first and last copies of the array are shown (blue box). Additional file 3: Table S1 provides the full list of the AGI numbers in between these two genes. The four single dispersed copies of the *SAT5* monomer containing different *CRP* genes or pseudogenes on chromosomes 2, 3, 4 and 5 are boxed in black. The pseudogenes on chromosome 2 (At2g19042) and chromosome 5 (At5g60978) were identified in the original MSAP analysis. The chromosome 4 gene (*CRP4*: At4g09545) has been analyzed in our study, which also considers two other genes in the CRP3600 subgroup that are not embedded in a SAT5 repeat (*CRP5a*: At5g42895 and *CRP5b*: At5g44495) (boxed in red). The single member of the CRP3600 subgroup containing introns (Atg53905) is indicated by the arrow. Additional information on CRP3600 subgroup members is given in Additional file 3: Figure S1. The figure was generated using the Chromosome Map Tool on the TAIR website http://www.arabidopsis.org/jsp/ChromosomeMap/tool.jsp..

Although *SAT5* was originally thought to be a non-protein coding repeat, improvements in the annotation of the *Arabidopsis* genome sequence during the course of our study revealed that genes encoding small cysteine-rich peptides (CRPs) and related *CRP* pseudogenes are embedded in the *SAT5* repeat monomer (Additional file 2: Figure S2). The *Arabidopsis* genome contains 825 *CRP* genes and pseudogenes that have been placed into different homology subgroups [26]. The particular subgroup associated with the *SAT5* repeat has been termed CRP3600, which has 57 members in *Arabidopsis*[26]. The CRP3600 subgroup has also been referred to as the DUF1278 family [27] and ECA1 gametogenesis-related proteins [28]http://www.arabidopsis.org. In addition to 32 copies embedded in the block of *SAT5* repeat monomers close to the centromere on chromosome 5, there are an additional 25 single copies of the CRP3600 family - not all of which are embedded in a *SAT5* repeat monomer-distributed on four out of the five *Arabidopsis* chromosomes (Figure 1; Additional file 3: Table S1). Eighteen of these are predicted to encode proteins, including a gene we have termed *CRP4* (At4g09545), which is embedded in a truncated, intergenic copy of *SAT5* on chromosome 4 (Table 1; Additional file 1: Figure S1). The remaining seven CRP3600 family members are likely pseudogenes, including two embedded in truncated *SAT5* copies on chromosomes 2 and 5, respectively, which were identified in the MSAP analysis (At2g18042 and At5g60978) (Table 1; Additional file 3: Table S1).

### Methylation of CRP coding regions: Pol IV/Pol V-dependent CHH methylation

**Figure 2:**
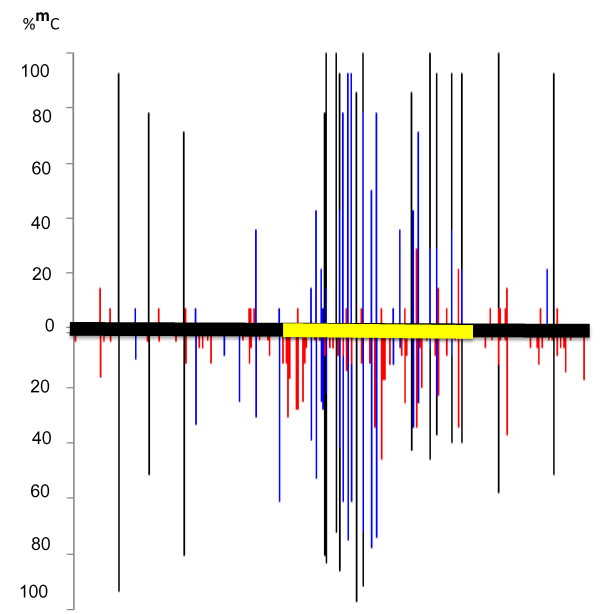
**DNA methylation of*SAT5*repeat monomer containing*CRP4*gene**. Bisulfite sequence analysis of DNA methylation of the *SAT5* repeat monomer containing the *CRP4* gene (At4g09545) in wild-type plants (bottom) and an *nrpe1 dcl3* double mutant (top). Methylation occurs in all three sequence contexts (CG, black; CHG, blue; CHH, red) and is concentrated in the *CRP4* coding region (yellow bar). Primarily CHH methylation is reduced in the *nrpe1 dcl3* double mutant. In a few cases, blue and black bars appear to be overlapping. These represent adjacent CG and CHG nucleotides that are not resolved in the figure format. CG methylation is always higher than CHG methylation.

To study methylation of the *SAT5* repeat, we performed a bisulfite sequence analysis on the truncated *SAT5* monomer containing the *CRP4* gene (1012 bp; Table 1). This sequence contains 369 bp *CRP4* coding region as well as 430 bp upstream and 213 bp downstream sequences (Additional file 2: Figure S2 ). Given its repetitive nature, we expected to detect heavy methylation throughout the *SAT5* sequence. We were surprised, therefore, to find non-uniform methylation that appeared to be concentrated within the *CRP4* coding region (Figure 2). One reason for this non-uniform distribution of methylation is that the number of cytosines available for methylation in CG and CHG sequence contexts differed along the length of the *SAT5* repeat. The numbers were relatively low in the regions upstream (3 CGs, 6 CHGs) and downstream (2 CGs, 1 CHG) but considerably higher in the *CRP4* coding region (11 CGs, 20 CHGs) (Table 2). Because at least some methylation was detected at all of the CGs and CHGs throughout the *SAT5* repeat monomer (Table 2), the density of CG and CHG methylation was naturally higher within the *CRP4* coding region compared to the flanking regions.

**Table 2:** Percent methylation of individual cytosines in SAT5 repeat monomer containing the *CRP4* gene

**Col-0**	methylated CG	unmethylated CG	methylated CHG	unmethylated CHG	methylated CHH	unmethylated CHH
upstream	3/3 (100 %)	0/3	6/6 (100 %)	0/6	22/51 **(43 %)**	29/51 (57 %)
CRP4 cds	11/11 (100 %)	0/11	20/20 (100 %)	0/20	44/52 **(85 %)**	8/52 (15 %)
downstream	2/2 (100 %)	0/2	0/1	1/1 (100 %)	19/28 **(68 %)**	9/28 (32 %)

The total number of cytosines and those that are methylated or unmethylated in each sequence context (CH, CHG, CHH) are shown for each region of the SAT5 repeat monomer containing the *CRP4* gene [upstream, CRP4 coding sequence (cds), downstream]. The percentage of methylated or unmethylated cytosines is shown in parentheses except where the value is 0 %. Percentages for methylated cytosines in a CHH context (indicative of RNA-directed DNA methylation) are shown in red. The difference for the upstream region vs. *CRP4* cds is significant (*p* = 0.000013; two-tailed Fisher’s exact test [http://www.graphpad.com/quickcalcs/index.cfm]). For the downstream region, the difference is not significant (*p* = 0.093) but this may reflect the lower number of cytosines in the downstream region.

**Figure 3:**
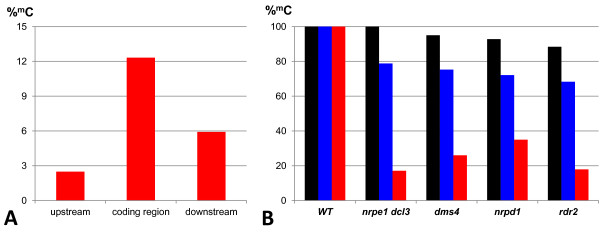
**CHH methylation in*CRP4*coding region. A**) Average percent methylation of cytosines in a CHH context in different parts of the *SAT5* repeat monomer containing the *CRP4* gene. CHH methylation is highest in the *CRP4* coding region. **B**) Overall levels of methylation in CG (Black), CHG (blue) and CHH (red) nucleotide groups in the *CRP4* coding region in wild type plants (WT) and mutants defective in RNA-directed DNA methylation (*nrpe1 dcl3* double mutant and *dms4*, *nrpd1*, and *rdr2* single mutants). Primarily CHH methylation is lost in the mutants.

The second factor influencing the *SAT5* methylation pattern was the tendency for CHH trinucleotides to be more frequently and more highly methylated within the *CRP4* coding region than in flanking sequences. Although the number of CHH nucleotide groups was approximately the same in the upstream region and *CRP4* coding region (51 and 52, respectively), methylation was only detected at around 22 CHHs (~43 %) in the upstream region, which differed significantly from the 44 CHHs (~ 85 %) with at least some methylation in the *CRP4* coding region (Table 2). In the downstream region, methylation was detected at 19 out of 28 CHHs (~68 %) (Table 2). In addition, the overall level of CHH methylation in the *CRP4* coding region was 12 % compared to ~ 3 % and ~ 6 % in the upstream and downstream regions, respectively (Figure 3A). Because CHH methylation is a hallmark of RNA-directed DNA methylation, these findings suggest that the *CRP4* coding region is targeted by this epigenetic pathway. Supporting this idea, the overall level of CHH methylation in the *CRP4* coding region was reduced ~ 65-80 % in various mutants defective in RNA-directed DNA methylation (Figure 3B). A similar pattern of dense CG, CHG and CHH methylation within the *CRP4* coding region together with considerably less methylation in the flanking regions was observed in the whole genome bisulfite sequencing (methylome) analysis by Lister and coworkers [8], http://neomorph.salk.edu/epigenome/epigenome.html.

The *CRP4* gene is not exceptional in its methylation pattern. Several other predicted protein-coding genes in the CRP3600 family, such as At3g42565 (embedded in a *SAT5* repeat; Table 1) and At5g42895 (*CRP5a*) and At5g44495 (*CRP5b*), which are not embedded in *SAT5* repeats (Additional file 3: Table S1), also contain in their coding regions dense CG, CHG and CHH methylation http://neomorph.salk.edu/epigenome/epigenome.html. This methylation is due in part to RNA-directed DNA methylation as evidenced by the loss of non-CG methylation in a *drm1 drm2 cmt3* (*ddc*) triple mutant http://neomorph.salk.edu/epigenome/epigenome.html, which isdefective in the *de novo* methyltransferase DRM2 that is required for RNA-directed DNA methylation [5,6].

### **Unique Pol IV-dependent 24-nt small RNAs originating from CRP coding regions**

**Figure 4:**
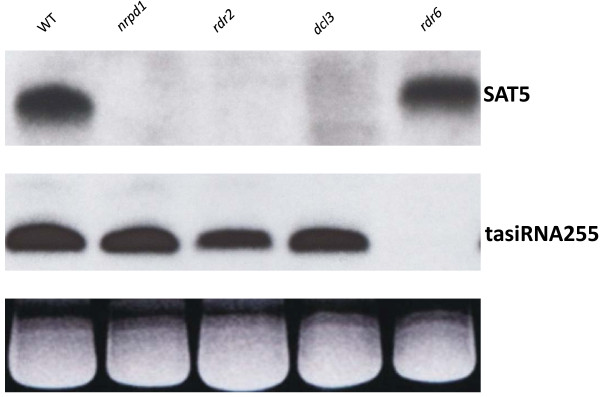
**Northern blot analysis of SAT5 small RNAs.** Bulk *SAT5* small RNAs that are 24-nt in length were detected using a probe derived from the *SAT5* consensus sequence. These small RNAs are present in wild-type plants (WT) as well as an *rdr6* mutant, which is defective in post-transcriptional gene silencing involving 21-nt small RNAs. The *SAT5* 24-nt small RNAs are undetectable in mutants defective in the Pol IV pathway (*nrpd1*, *rdr2*, *dcl3*). The blot was re-probed with a probe for a trans-acting small RNA (tasi255), which requires RDR6 for its biogenesis and is independent of Pol IV pathway components. The major RNA on the ethidium bromide stained gel is shown as a loading control (bottom).

**Figure 5:**
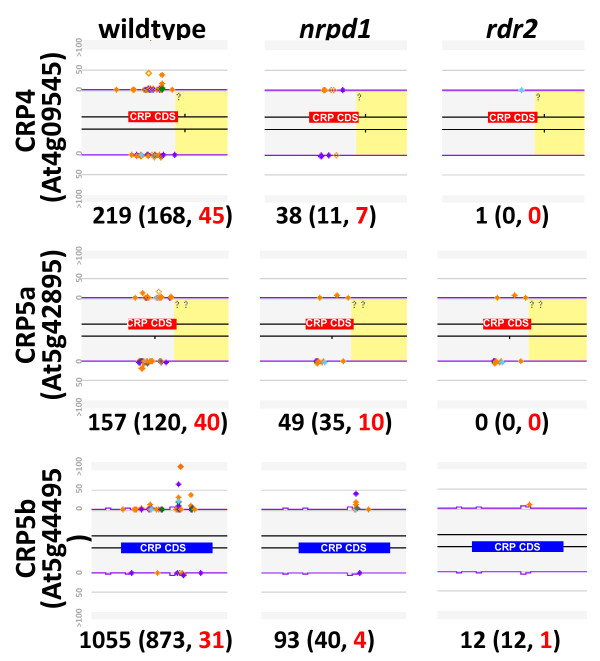
**Small RNAs originating from specific*CRP*coding regions.** Individual abundances for small RNA signatures between 21–24 nt in length are displayed in different colors (24-mers are orange). A unique small RNA signature is displayed as a filled diamond and a duplicated small RNA signature is displayed as a hollow diamond. The exons of *CRP4*, *CRP5a* and *CRP5b* genes are annotated as blue or red boxes. Purple lines indicate a k-mer frequency for repeats; yellow shading indicates DNA transposon-like sequences found by RepeatMasker. Note that the majority of small RNAs originate within the *CRP* coding regions and not in the adjacent transposon-like sequences. The small RNA abundances were normalized to 5 transcripts per million (TPM). The sum of hits-normalized-abundance (HNA) for all small RNAs mapping to the entire gene is displayed below each gene image with the total abundance of 24 nt siRNAs in parentheses in black, followed by the count of unique 24 nt siRNAs in red text.

Our bisulfite sequencing analysis and the independent methylome analysis of Lister and coworkers [8]http://neomorph.salk.edu/epigenome/epigenome.html suggested that methylation of the coding regions of *CRP4CRP5a* and *CRP5b* genes is due in part to RNA-directed DNA methylation. We therefore tested whether the requisite Pol IV-dependent 24-nt small RNAs needed to induce RNA-directed DNA methylation originate from these sequences. A Northern blot analysis using a *SAT5* probe readily detected 24-nt small RNAs that disappeared in Pol IV pathway mutants (*nrpd1rdr2dcl3*) (Figure 4). Because the *SAT5* probe detected bulk small RNAs originating from this repeat, we used deep sequencing to investigate in more detail small RNAs arising from individual *CRP* genes. This analysis indeed revealed unique 24-nt small RNAs associated with the coding regions of the *CRP4CRP5a* and *CRP5b* genes and these small RNAs were reduced in Pol IV pathway mutants (*nrpd1* and *rdr2*) (Figure 5).

### **Expression of CRP genes in the female gametophyte**

**Figure 6:**
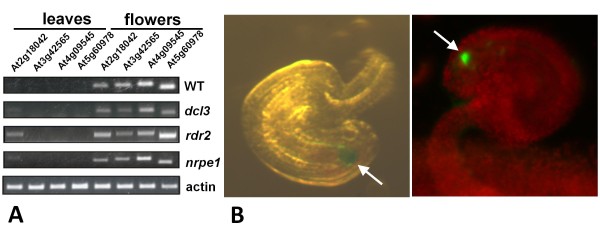
**Flower and synergid cell-specific expression of*CRP*genes and the*CRP4*promoter.** (**A**) Semi-quantitative RT-PCR demonstrates expression of endogenous *CRP* genes (At3g49565, At4g09545) and *CRP* pseudogenes (At2g18042, At5g60978) in flowers but not in leaves. With the exception of the pseudogene At2g18042, which shows some reactivation in leaves in mutants defective in RNA-directed DNA methylation (*dcl3*, At3g43920; *rdr2*, At4g11130; *nrpe1*, At2g40030), none of the genes or pseudogenes are appreciably expressed in these mutants. Actin (At3g18780) is shown as a constitutively expressed control. (**B**) Photographs of ovules from transgenic *Arabidopsis* plants transformed with constructs containing *CRP4-GFP* (right) and *GUS* (left) reporter genes under the control of the *CRP4* promoter. Expression occurs exclusively in the synergid cell region of the female gametophyte (arrows). Identical results were obtained in multiple independent transgenic lines obtained for each construct.

A previous genome-wide expression profiling analysis demonstrated that many *CRP* genes in the CRP3600 subgroup, including *CRP5a* and *CRP5b*, are expressed in the egg apparatus (egg and synergid cells) of the female gametophyte [27]. However, the expression patterns of the four CRP3600 subgroup members embedded in single copy *SAT5* repeat monomers (two genes and two pseudogenes) (Table 1) has not yet been reported. Using semi-quantitative RT-PCR, we detected transcripts from these two genes and two pseudogenes in flowers but not leaves, and with the exception of the pseudogene *At2g18042*, transcription is not strongly reactivated in mutants defective in RNA-directed DNA methylation (Figure 6A).

To obtain a more detailed view of the cell type-specific expression pattern, we generated constructs encoding a *GUS* reporter protein and a *CRP4-GFP* fusion protein, both under the control of a *CRP4* promoter fragment, which comprises 921 bp upstream of the ATG start codon of the *CRP4* gene. After introducing these constructs into wild-type *Arabidopsis* plants, *GUS* and *CRP4-GFP* gene expression was observed specifically in the synergid cell region of the female gametophyte, indicating that the *CRP4* promoter is active exclusively in these cells (Figure 6B). We therefore infer that the detection of *CRP4* transcripts in flowers of non-transgenic plants (Figure 6A) reflects expression of the endogenous *CRP4* gene in synergid cells.

### **CRP4 coding sequence is not methylated when transcribed by Pol II to produce a translatable mRNA**

**Figure 7:**
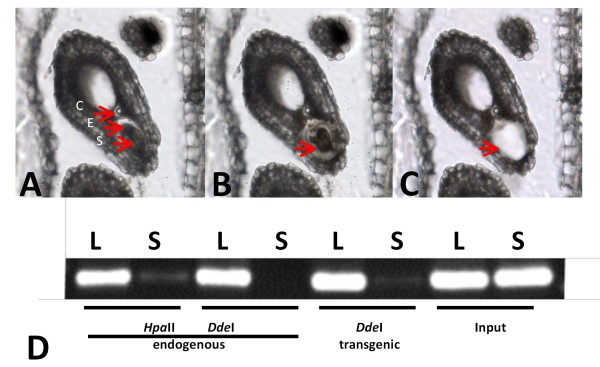
**Laser-Assisted Microdissection and subsequent DNA methylation analysis of*CPR4*sequences in synergid cells from mature embryo sacs.** (**A**) Dissection of the synergid cell from a mature embryo sac; an 8 μm section through an ovule bearing a mature embryo sac prior to laser microdissection with the MMI CellCut Plus instrument. The following abbreviations are used: S, synergids; E, egg cell; C, central cell. (**B**) The ultraviolet laser beam has been applied in order to isolate the synergid cells (arrow). The laser cut has a diameter of 1–2 μm. (**C**) The synergid cell has been removed with an MMI isolation cap. (**D**) DNA methylation analysis. Cytosine methylation of the *CRP4* coding sequence was studied in synergid cell (S) and leaf genomic DNA (L), in both endogenous and transgenic contexts, using enzymes sensitive to CG/CHG methylation (*HpaII*) and CHH methylation (*DdeI*). The *CRP4* coding region has two sites for *HpaII* and three sites for *DdeI*. Disappearance or reduced levels of a fragment after digestion with a given enzyme indicate loss of methylation at that site. Undigested input DNA is included at the far right.

**Figure 8:**
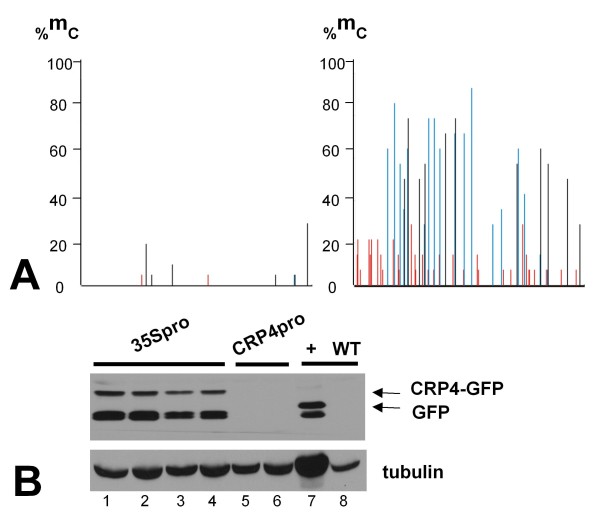
***CRP4*coding sequence is unmethylated in leaves when transcribed from 35S promoter to produce translatable mRNA.** (**A**) Bisulfite sequencing to assess methylation in leaves of the *CRP4* coding region in a *CRP4-GFP* fusion gene under the control of the constitutive 35S promoter (left) and the synergid cell-specific *CRP4* promoter (right). The region shown corresponds to that indicated by the yellow bar in Figure 2. We also performed bisulfite sequencing on the *GFP* coding sequence in the *CRP4* promoter-directed construct but observed no methylation (data not shown). (**B**) Western blot detection of the CRP-GFP fusion protein in seedlings from four independent transgenic lines transformed with a 35S promoter-*CRP4-GFP* fusion construct (lanes 1–4, top band, top arrow). The ‘+’ lane shows control GFP expression in a previously published transgenic line (top band, bottom arrow) [23,24]. The lower band in these lanes probably corresponds to a degradation product that reacts with the GFP antibody. A CRP4-GFP fusion protein is not detected in transgenic plants containing a *CRP4-GFP* fusion gene under the control of the *CRP4* promoter (lanes 5 and 6; results from two independent lines shown). Tubulin is shown as a constitutively expressed control.

To study methylation of the *CRP4* coding region in cells where the *CRP4* promoter is active, we isolated DNA from synergid cells of a transgenic plant expressing a *CRP4-GFP* fusion gene under the control of the *CRP4* promoter. A PCR-based approach was used to examine methylation in the *CRP4* coding sequence of this fusion gene as well as the endogenous *CRP4* gene. For this, DNA was digested with either *Hpa*II (reporting on CG methylation) or *Dde*I (reporting on CHH methylation), and PCR amplification was performed using primers specific for the *CRP4* coding sequence in either the endogenous *CRP4* gene or the *CRP4-GFP* fusion gene. In both cases, little or no amplification product was observed when using synergid cell DNA, indicating reduced methylation of the endogenous and transgenic *CRP4* coding sequences in cells where the *CRP4* promoter is active (Figure 7). By contrast, a strong amplification product indicating the presence of DNA methylation was seen for both the endogenous and transgenic *CRP4* sequences when using leaf DNA (Figure 7). The latter results are consistent with the bisulfite sequence analysis on leaf DNA, which revealed dense methylation in the *CRP4* coding region of both the endogenous *CRP4* gene (Figure 2) and the *CRP4* promoter-driven *CRP4*-*GFP* fusion (Figure 8A, right).

We obtained further evidence that the *CRP4* coding sequence is unmethylated when transcribed by Pol II by examining methylation of a *CRP4-GFP* fusion gene under the control of the constitutive 35 S promoter in leaves. In transgenic plants containing this construct, the *CRP4* coding sequence of the *CRP4-GFP* fusion gene remained largely unmethylated (Figure 8A, left). Moreover, a CRP4-GFP fusion protein was detectable on Western blots in these plants, indicating Pol II transcription of a translatable *CRP4-GFP* mRNA from the 35 S promoter (Figure 8B, lanes 1–4). By contrast, the *CRP4* coding sequence of a *CRP4-GFP* fusion gene under the control of the *CRP4* promoter acquired methylation similar to the endogenous *CRP4* gene (Figure 8A, right) and no CRP4-GFP fusion protein was detected in plants containing this construct (Figure 8B, lanes 5,6). Thus, *CRP4* coding sequences transcribed by Pol II lack methylation in both synergid cells, where the endogenous *CRP4* gene and *CRP4-GFP* fusion gene are expressed from the *CRP4* promoter, and in leaves, where the fusion gene is under the control of the constitutive 35S promoter.

### The CRP4 gene is not inducible by flg22 or heat treatment in seedlings

**Figure 9:**
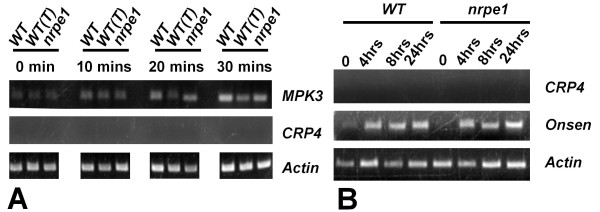
**The*CRP4*gene is not induced in seedlings following flg22 treatment or heat stress.** Semi-quantitative RT-PCR was used to test possible induction of *CRP4* gene expression in seedlings under different conditions. (**A**) Treatment of seedlings with the bacterial elicitor flg22 for the indicated time periods results in induction of expression of the MAP kinase *MPRK3*[31] but not the *CRP4* gene. Similar results were seen in wild-type non-transgenic seedlings (WT), a wild-type transgenic line [WT(T)], and an *nrpe1* mutant, which is defective in the largest subunit of Pol V. (**B**) Heat treatment of seedlings for the indicated time periods does not result in induction of *CRP4* gene expression whereas the *Onsen* retrotransposon is induced under the same conditions [19]. In both panels, actin expression is shown as a constitutively expressed control.

The endogenous *CRP4* gene was not de-repressed in the leaves of mutants defective in RNA-directed DNA methylation (Figure 6A) but it is possible that biotic or abiotic stresses might reactivate the *CRP4* gene. Given the suggested roles of CRPs in microbial defense [29], we tested whether the *CRP4* gene was induced after treatment of seedlings with the bacterial flagellin peptide flg22, which is a potent elicitor of the innate immune response in plants [30]. Although we observed induction of MPK3 (*At3g45640*), a known flagellin-induced gene [31], no induction of the *CRP4* gene was observed under the same conditions (Figure 9A). Similarly, we did not observe induction of the *CRP4* gene after heat stress that was sufficient to activate the *Onsen* retrotransposon (Figure 9B), which requires Pol IV pathway components to restrict trans-generational transposition [19].

## **Discussion**

Current views of DNA methylation patterns in plants hold that gene body methylation occurs primarily in a CG context and that non-CG methylation is limited to transposons and non-protein-coding repeats [13,32]. Contrary to this view, we have identified several genes encoding CRPs in *Arabidopsis* that atypically contain in their gene bodies not only CG but also CHG and CHH methylation, which arises in part from RNA-directed DNA methylation. These genes are thus methylated like transposons in their coding regions. Evidence for this claim includes the detection of Pol IV-dependent 24-nt small RNAs that are unique to each gene and the reliance of full CHH methylation on components of the Pol V pathway. In addition to RNA-directed DNA methylation, other methylation pathways contribute to the dense methylation of *CRP* gene bodies in leaves. This is indicated by the persistence of nearly wild-type levels of CG and CHG methylation in mutants defective in RNA-directed DNA methylation, which lose predominantly CHH methylation. Multiple layers of epigenetic modifications have been observed previously for other targets of RNA-directed DNA methylation in plants [13,15,32,33]. The biological role of the transposon-like methylation pattern of *CRP* gene bodies is not yet clear but it is likely to be related to gene silencing in the sporophyte. As discussed below, alternative functions can also be considered.

The *CRP* genes investigated in this study are silent and methylated in leaves and, given the restricted expression pattern of the *CRP4* promoter when fused to *GUS* and *GFP* reporter genes, presumably in other sporophytic tissues as well. As shown here and elsewhere [27], the promoters of many *CRP* genes in the CRP3600 family are active specifically in the egg apparatus region of the female gametophyte (i.e. egg and synergid cells). The functions of CRPs in the female gametophyte are not yet clear but possibilities include acting as pollen tube attractants [34], antimicrobial substances in the vulnerable egg cell region [29,35,36], or intercellular signaling molecules during female gametophyte development [37-39]. In our experiments, the synergid cell-specific expression of the *CRP4-GFP* fusion gene, and presumably the *CRP4* endogenous gene, was correlated with a loss of CG and CHH methylation in the *CRP4* gene body at the restriction sites tested. This finding suggests a connection between the observed gene body methylation and *CRP4* gene silencing in leaves. We assume a similar association holds true for the *CRP5a* and *CRP5b* genes, which are highly methylated in their gene bodies in leaf tissue http://neomorph.salk.edu/epigenome/epigenome.html yet expressed exclusively in the egg apparatus region of the female gametophyte [27].

The influence of gene body methylation on expression depends on the sequence context of methylated cytosines. In wild-type plants, CG methylation alone in gene bodies does not inhibit transcriptional elongation and is found at many moderately expressed genes [8,9,13,16]. CHG methylation in gene bodies, which is induced in the *ibm1* (*increase in BONSAI methylation1*) mutant, has variable effects on transcription, with only some target genes showing decreased transcription while others are unchanged or even more highly expressed [40]. By contrast, our results suggest that a combination of dense CG and non-CG methylation in gene bodies is associated with gene silencing. Whether this methylation inhibits transcriptional elongation by Pol II or is the default state when transcription is impaired by other means is not yet known. Also unclear is how methylation in *CRP4* gene bodies is lost in synergid cells. It may result from active demethylation by DNA glycosylase activities, as observed for imprinted genes in the central cell of the female gametophyte [41,42], or a lack of silencing factors in synergid cells or their precursors. A recent global enrichment study demonstrated that genes encoding components of the RNA-directed DNA methylation machinery, in particular six PAZ-domain proteins, are predominantly expressed in egg cells but some are also expressed in synergid cells [43]. Loss of *CRP4* gene body methylation in synergid cells may also reflect the restricted cellular distribution of essential transcription factors, such as the synergid cell-specific transcription factor MYB98 [27,44]. However, there does not seem to be a correlation between methylation of *CRP* genes in leaves and a requirement for MYB98 in synergid cell-specific expression. For example, both *CRP5a* and *CRP5b* are methylated in leaves and expressed in synergid cells but only the former requires MYB98 [27].

Although gene silencing seems the most likely function and/or consequence of combined CG and non-CG methylation in the *CRP* gene bodies, a possible positive role can also be considered, namely that gene body methylation attunes genes to internal or external cues. This suggestion follows from recent data demonstrating that gene body methylation, whether in a CG or non-CG context, can be dynamic and may render genes sensitive to developmental or environmental signals. For example, a bioinformatics study examining genome-wide gene expression in *Arabidopsis* under many different experimental conditions concluded that CG methylation in gene bodies protects genes from reacting to internal or external stimuli [18]. In tomato, a drought-responsive gene that atypically has non-CG methylation in the gene body loses non-CG methylation and gains CG methylation under drought conditions [45]. Some transcribed genes in human embryonic stem cells contain in their gene bodies non-CG methylation that is lost as cells differentiate and re-established during de-differentiation, suggesting a relationship between non-CG methylation in gene bodies and the differentiation state of the cells [14,46]. In view of these findings, it is conceivable that combined CG and non-CG methylation in *CRP* gene bodies allows responsiveness to either developmental cues in the female gametophyte or external factors in the environment. Regarding the latter possibility, we did not observe de-repression of the methylated *CRP4* gene in leaf tissue following treatment with flg22 or heat stress, but other biotic or abiotic stresses can be tested in the future.

Why are the gene bodies of some CRP3600 gene family members methylated like transposons? Although several methylated *CRP* genes are embedded in *SAT5* repeats this is not the case for all (for example, *CRP5a* and *CRP5b*), demonstrating that the presence of the flanking *SAT5* sequences is not a requirement for methylation. To explain the dense methylation concentrated in the *CRP* coding regions, we propose that some members of the CRP3600 gene family arose through reverse transcription of mature mRNAs (retroposition) and hence can be classified as retroposed genes (retrogenes). Under this hypothesis, an intron-containing progenitor copy would have been highly expressed in the egg cell or one of its precursors in the germline, the spliced mRNA reverse transcribed, and the cDNA subsequently integrated into egg cell DNA to become a heritable part of the genome. Through this copying and integration process, *CRP* retrogenes would resemble transposons and be methylated accordingly in their coding regions. Consistent with this hypothesis, only one gene in the CRP3600 family has two introns (At5g53905) while the rest are intron-free and thus candidate retrogenes (Table 2). The observation that at least some CRP3600 gene family members are probably expressed in the egg cell as well as the neighboring synergid cells [27] is compatible with the retrogene hypothesis. Interestingly, retrogenes in mammals are expressed mainly in the testes, where transcription is less tightly regulated than in somatic tissues [47]. Whether controls on transcription are similarly relaxed in synergid cells and/or egg cells in plants remains to be determined.

Not all copies of the CRP3600 gene family have a transposon-like methylation pattern in their gene bodies. Some lack methylation entirely and one has CG methylation only (Additional file 3: Table S1). Although the source of such differences is presently unknown, one possibility is that recently integrated copies still have the initial transposon-like methylation pattern, which over time degenerates into exclusively CG methylation, ultimately resulting in the complete decay of gene body methylation. Because methylated cytosine spontaneously deaminates to produce thymine, methylated *CRP* coding regions may experience accelerated sequence diversification over evolutionary time, thus promoting functional diversification of CRP proteins. Given known or proposed roles of CRPs in pollen tube guidance [34] and self-incompatibility [38,48], such sequence changes and functional diversification may help to drive speciation.

## **Conclusions**

We have identified several genes of the *Arabidopsis* CRP3600 subgroup that have an unusual transposon-like methylation pattern consisting of CG, CHG and CHH methylation in their gene bodies. This methylation, which was observed in leaves, appears to be associated with gene silencing in sporophytic tissues because it decreases in synergid cells of the female gametophyte, where the *CRP* genes are specifically expressed. Analysis of CRP3600 gene family members suggests that at least some methylated copies may have arisen through retroposition, causing them to be epigenetically modified like transposons through RNA-directed DNA methylation and other methylation pathways that target mobile elements. The *CRP* genes will be useful models for studying developmentally-regulated changes of combined CG and non-CG methylation in gene bodies and the expansion through retrotransposition of gene families expressed in the plant germline.

## **Methods**

### **Plants**

All experiments have been performed using *Arabidopsis thaliana* accession Col-0. For the mutants defective in RNA-directed DNA methylation the alleles used were *drd3-7* (for *nrpe1*) [23], *rdr2-1* (SAIL_1277H08; [49], *nrpd1-7* (formerly *nrpd1a-7*; [50]), *dms4-1*[51], *dcl3-1* (SALK_005512; [49]) and *rdr6-1*[52]. Mutants were genotyped using primers shown in Additional file 4: Table S3. Plants were cultivated on potting soil in either a greenhouse or growth chamber set at ~ 21°C with a 16 hour light/8 hour dark cycle.

### **Constructs and plant transformation**

For expression studies, three constructs were made: 35Spromoter-*CRP4-EGFPCRP4*promoter-*CRP4-EGFP*, and *CRP4*promoter-*GUS*. To assemble the constructs, the *CRP4* promoter region (consisting of 921 bp upstream of ATG start codon) and the *CRP4* coding region (369 bp) were modified with restriction enzyme sites that are necessary for the plasmid construction [modified sequence synthesized by Mr.Gene (Regensburg, Germany)]. For construction of the *CRP4-GFP* fusion gene, a sequence encoding enhanced green fluorescent protein (EGFP, referred to hereafter as GFP) was added to the C-terminus of *CRP4* (including both promoter and coding region). We also fused the same *GFP* coding sequence to the C-terminus of the *CRP4* coding region, which was driven by the cauliflower mosaic virus 35 S promoter. Both of these fragments were inserted into a pPZP221 binary vector [53], which has a gentamicin selection marker driven by the 35 S promoter [54] for selection of transformed plants. To construct the ß-glucuronidase (GUS) reporter under the control of *CRP4* promoter, a *GUS* coding sequence (plasmid pRT102) [55] was inserted downstream of the *CRP4* promoter and introduced into pPZP221 binary vector [53].

All three plasmids (pPZP 35Spromoter-*CRP4-GFP*, pPZP *CRP4* promoter-*CRP4-GFP*, and pPZP *CRP4*promoter-*GUS* ) were transferred into *Agrobacterium tumefaciens* strain ASE using triple mating [56] and transgenic plants were obtained by using the floral dip method [57].

### **Methylation-sensitive amplification polymorphism (MSAP)**

MSAP analysis was performed according to a published procedure [21] with some modifications. Genomic DNA was isolated using the Qiagen DNAeasy Maxi Kit (Qiagen, Hilden, Germany) and 1 μg of genomic DNA was digested with *EcoT22*I/*Mse*I overnight. Fifty pMol *Mse*I adapters and 5 pMol *Pst*I adapters were added and ligation was performed for 3 hrs at 37°C. Sterile H2O up to 500 μl was added and the samples were stored at - 20°C until use. Adapters were prepared by adding equimolar amounts of both strands.

For PCR reactions, the *Eco*T22I primer was radioactively labeled using [γ^32^P] ATP and T4 polynucleotide kinase (reaction carried out on ice) (Roche, Vienna, Austria). The following PCR program was used: 1 cycle: 1 minute 94°C, 30 seconds 65°C, 1 minute 72°C afterwards the annealing temperature was lowered each cycle by 0.7°C during 12 cycles. This gave a touch down phase of 13 cycles. This was followed by: 23 cycles: 30 seconds 94°C, 30 seconds 56°C, 1 minute 72°C; 10 minutes 72°C; ∞ 4°C. PCR products were electrophoresed on 5 % polyacrylamide-7.5 M urea-gels [acrylamide:bis-acrylamide, 19:1, (Roth, Karlsruhe, Germany]. Pre-electrophoresis was run at 60 Watt for 30 minutes in 1 x TBE running buffer until plates were at approximately 45°C. Electrophoresis was performed for 2 hours 30 minutes at 60 Watt until xylene cyanol was two-thirds down of the length of the gel. Gels were blotted onto 3MM Whatman filter paper covered with Saran Wrap and dried in the gel dryer for 2 hours 30 minutes at 80°C. The gels were exposed to Kodak BioMax MS Film overnight at room temperature.

Bands showing polymorphisms were cut out from the 3MM Whatman filter paper. 100 μl sterile H_2_O was added and the bands were incubated o/n at 4°C. DNAs were reamplified and cloned into the pGEM-T Easy vector (Promega, Vienna, Austia). The following PCR program was used for re-amplification: 95°C 2 minutes; 95°C 10 seconds, 56°C 20 seconds, 72°C 1 minute 30 seconds, 35 cycles; 72°C 7 minutes. Cloned fragments were re-run on an MSAP gel side by side with the genomic DNA fragments to make sure that the correct fragment was cloned and to check the reproducibility of the polymorphism. Robust polymorphic bands were sequenced.

Primers used to detect and clone the chromosome 2 *SAT5* copy were T21 (5′-GAC TGC GTA GGT GCA TCC-3′) and M56 (5′-GAT GAG TCC TGA GTAATG T-3′). For the chromosome 5 *SAT5* copy, the primers used were T21 (see above) and M1 (5′GAT GAG TCC TGA GTA ATA-3′).

### **Bisulfite sequencing**

Genomic DNA was isolated from rosette leaves using a DNeasy Plant Maxi kit (Qiagen, Hilden, Germany). Bisulfite treatment of DNA was conducted using a EpiTect Bisulphite kit (Qiagen, Hilden, Germany) according to the manufacturer’s instructions with several modifications as described previously [58]. The PCR reactions were performed using Advantage 2 Polymerase Mix from Clontech (Mountain View, California) and the conditions for the amplification of bisulfite-treated DNA were as follows: 94°C for 2 min followed by 34–39 cycles at 94°C for 30 sec, 30 sec annealing temperature for a particular primer pair (see Additional file 4: Table S3), 68°C for 45 sec, and 6 min of final elongation. The PCR was carried out in a total reaction volume of 50 μl. As a control for complete bisulfite conversion, we used the PHAVOLUTA gene [59]: PCR conditions are the same as above except for the first pair of primers we used 40 cycles and for the second pair 35 cycles. Primers are shown in Additional file 4: Table S3. The 1012 bp *SAT5* copy containing the *CRP4* gene was analyzed in three overlapping parts. The results shown in Figure 2 are from sequencing of at least 15 clones of each part in wild-type and mutant plants.

### **Small RNA isolation and Northern blot analysis**

Small RNAs were isolated from pooled 21-day-old *Arabidopsis* seedlings or inflorescence tissues using the mirVana miRNA isolation kit (Ambion/Applied Biosystems, Brunn am Gebirge, Austria) and analysed by Northern blot hybridization according to a published procedure [23,33,58]. We used an end-labeled oligonucleotide probe to detect tasiRNA255 (5′-TAC GCT ATG TTG GAC TTA GAA-3′) and an end-labeled LNA (locked nucleic acid) oligonucleotide (5′-AAL CAA LAA TLG TTL GGT LCA TLC CGL TGA LTG GLA TAL TTC LCT GLG ATL GCA-3′ as a probe to detect bulk *SAT5* small RNAs.

### **Small RNA sequencing**

Libraries of small RNAs were constructed from total RNA isolated from mixed stage inflorescence tissues of wild type (wt) *Arabidopsis* Col-0, mutants *nrpd1* and *rdr2* using TRIzol reagent (Invitrogen). Total RNA (200 μg) for each sample was used to construct the small RNA librarires as described previously but with different adapters. The RNA oligos (Dharmacon) used for small RNA ligations were as follows: 5′ RNA Adapter (5′OH-GUUCAGAGUUCUACAGUCCGACGAUC-OH 3′) and 3′ RNA Adapter: (5′ pUCGUAUGCCGUCUUCUGCUUGUidt 3′; p, phosphate; idT, inverted deoxythymidine). Libraries were sequenced on an Illumina HiSeq2000 at the Delaware Biotechnology Institute. This generated 12,043,425 total reads from wt Col-0, 13,580,575 from mutant *nrpd1* and 9,001,733 from mutant *rdr2*.

Adapter sequences were removed using a Perl script, generating small RNA sequences plus abundances. The data were matched to the Arabidopsis genome (TAIR v9). Of the 12,043,425 total reads from wt Col-0, 13,580,575 from mutant *nrpd1* and 9,001,733 from mutant *rdr2*, 8,577,577 (wt), 2,858,557 (*nrpd1*) and 4,322,052 (*rdr2*) reads matched the genome, excluding 1,709,653 (wt), 8,889,937 (*nrpd1*) and 3,664,258 (*rdr2*) that matched tRNA, rRNA, snRNA or snoRNAs. Libraries of small RNAs were constructed from total RNA isolated from mixed stage inflorescence tissues of wild type (WT) Arabidopsis Col-0, mutants *nrpd1* and *rdr2* using TRIzol reagent (Invitrogen, Carlsbad, CA, USA). Total RNA (200 μg) for each sample was used to construct the small RNA libraries as described previously [60] but with different adapters. The RNA oligos (Dharmacon, Lafayette, Colorado, USA) used for small RNA ligations were as follows: 5′ RNA Adapter (5′OH-GUUCAGAGUUCUACAGUCCGACGAUC-OH 3′) and 3′ RNA Adapter: (5′ pUCGUAUGCCGUCUUCUGCUUGUidt 3′; p, phosphate; idT, inverted deoxythymidine). Libraries of small RNAs were sequenced using Illumina’s (San Diego, CA, USA) sequencing by synthesis technology at the Delaware Biotechnology Institute. This generated 12,043,425 total reads from wt Col-0, 13,580,575 from mutant *nrpd1* and 9,001,733 from mutant *rdr2*.

Adapter sequences were removed using a Perl script, generating small RNA sequences plus abundances. The data were matched to the Arabidopsis genome (TAIR v9). Of the 12,043,425 total reads from wildtype Col-0 (WT), 13,580,575 from mutant *nrpd1* and 9,001,733 from mutant *rdr2*, 8,577,577 (WT), 2,858,557 (*nrpd1*) and 4,322,052 (*rdr2*) reads matched the genome, excluding 1,709,653 (WT), 8,889,937 (*nrpd1*) and 3,664,258 (*rdr2*) that matched tRNA, rRNA, snRNA or snoRNAs. The small RNA sequence data are available from NCBI's Gene Expression Omnibus (GEO) and are accessible via GEO Series accession number GSE34207. Accession numbers for the separate libraries are GSM844489 (rdr2_sRNA); GSM844490 (nrpd1 rdr2_sRNA); and GSM844491 [WT (Col-0)_sRNA].

### **Semi-quantitative RT-PCR**

Total RNA was extracted from 3 week old seedlings (100 mg) or mixed floral inflorescences (100 mg) by using TRIzol® Reagent (Invitrogen, Lofer, Austria) according to the manufacturer’s instructions. One μg of total RNA was used for reverse transcription using RevertAid™ H Minus First Strand cDNA Synthesis Kit (Fermantas, St. Leon-Rot, Germany) following the manufacturer’s instructions. One μL cDNA was used for semi-quantitative reverse transcriptase–mediated (RT) PCR analysis. The PCR conditions were 95°C for 5 min followed by 23 (actin), 25 (MPK3), 40 (*CRP* genes) amplification cycles (95°C for 30 s, 55°C for 30 s, and 72°C for 1 min). Actin (At3g18780) was used a constitutively expressed internal control. Primers used for RT-PCR are listed in Additional file 4: Table S3.

### **GUS assays and GFP visualization**

Transgenic plants transformed with the three constructs described above were used to detect synergid cell-specific expression of *GUS* and *GFP* reporter genes. GUS assays were performed as described previously [28]. Briefly, ovules were dissected from early opened flowers (emasculated 24 hr before) in a small petri dish containing GUS staining solution (50 mM sodium phosphate buffer pH 7.0, 10 mM EDTA, 0.1 % Triton X-100, 2 mM potassium ferrocyanide, 2 mM potassium ferricyanide, and 1 mg/ml X-Gluc) on ice. The ovules were incubated at 37°C for 30 min, cleared with 20 % methanol/4 % concentrated HCl solution at 55°C for 15 min, and then transferred into a solution containing 60 % ethanol/1.8 M NaOH at 25°C for 10 min. Samples were washed with 30 % ethanol and 10 % ethanol and mounted onto slides with a 50 % glycerol solution. Images were acquired using a Leica stereofluorescence microscope MZ16FA equipped with a Leica GFP2 filter set and a Leica DFC 300FX camera (Leica GmbH, Germany).

To visualize GFP, ovules were dissected from early opened flowers (before fertilization), and localization of the GFP signal was viewed by using Leica stereofluorescence microscope MZFLIII.

### **Tissue Embedding and Laser-Assisted Microdissection (LAM)**

Flowers were emasculated and harvested 48–72 hrs thereafter. The tissue was fixed in EtOH:Acetic Acid 3:1 (v/v) under vacuum for 2x 15 min at 4°C, according to Kerk and coworkers [61], then left in the fixative overnight at 4°C. The tissue was embedded in Paraplast® Regular embedding media (Sigma-Aldrich, Vienna, Austria) using an automated protocol.

In the automated procedure, the fixed tissue was embedded using the automated Sakura Tissue-Tek®VIP™ embedding machine (Sakura Finetek Europe B.V., Alphen aan den Rijn, The Netherlands) with the following parameters: 1 hr 70 % EtOH at 37°C, 1x 1 h 80 % EtOH, 2x 1 h 96 % EtOH, 2x 1 h 100 % EtOH, 2x 1 h 100 % Xylol, all at 40°C, and then 4x 1 h Paraplast® Regular embedding media (Sigma-Aldrich, Vienna, Austria) at 60°C. Flowers were poured into paraffin-blocks, cooled and then kept at 4°C until use.

For microdissection, the embedded flowers were sectioned on the Leica microtome RM2145 (Leica Microsystems GmbH, Wetzlar, Germany) to 8 μm thickness and mounted onto RNase-free membrane slides (Molecular Machines & Industries [MMI] AG, Glattbrugg, Switzerland) with the use of RNAse free miliQ water. Slides were dried overnight on a heating table at 42°C and processed the following day. Microdissection was performed with a CellCut Plus instrument (MMI AG, Glattbrugg, Switzerland), which makes use of a solid state UV-A laser (wavelength approx. 350 nm) to cut the tissue. Each slide was deparaffinized by processing in 100 % Xylene for 2x 10 minutes at room temperature. Slides were air-dried for 10 min. and immediately used for laser microdissection. Synergids cells were harvested using MMI isolation caps, with an average of 110 cells isolated per cap. Due to the cytology of the egg apparatus, it was not possible to isolate the synergids cells without including a small basal part of the egg cell in the same section [43]. However care was taken to avoid contamination of the sample with egg cell nuclei. Therefore, for DNA methylation studies the sample can be considered as purely synergid cells.

### **Detection of CRP4-GFP fusion proteins by Western blotting**

Total proteins were extracted from 3 week old seedlings grown on solid Murashige and Skoog (MS) medium. Approximately 50 mg seedlings were harvested, ground in liquid nitrogen and resuspended using 100 μl extraction buffer (50 mM HEPES-KOH pH 7.9, 400 mM KCl, 2.5 mM MgCl_2_, 1 mM EDTA, 1 mM DTT, 0.1 % Triton X-100) supplemented with EDTA-free protease inhibitor cocktail (Roche, Vienna, Austria). After centrifuging 15 min at 4°C, the supernatant was mixed with the same volume of extraction buffer without KCl. Proteins were separated using 10 % SDS–PAGE and transferred to a PVDF membrane (Millipore, Vienna, Austria). Membranes were probed either with mouse monoclonal antibodies against GFP (1:1,000) (Roche, Vienna, Austria) or tubulin (1:1,000) (Sigma-Aldrich, Vienna, Austria), followed by horseradish peroxidase conjugated goat anti-mouse secondary antibody (1:10,000, Biorad, Vienna, Austria). The blots were developed using an enhanced chemoluminescence ECL kit (Thermo Scientific, Vienna, Austria), and exposed to CL-XP X-ray film (Thermo Scientific, Vienna, Austria).

### **Treatment with flg22 and heat stress**

Dried seeds were sterilised with 70 % ethanol/ 0.05 % tritonX-100 and sown on solid MS medium. One week after germinating, small seedlings were transferred to liquid MS medium supplied with 1 μM flg22 peptide (kindly provided by Elke Logemann and Imre Somssich, Max Planck Institute for Plant Breeding Research, Cologne) (4 seedlings per 400 μl of medium in wells of 24 well plates). Batches of seedlings were collected after 10 min, 20 min, and 30 min of flg22 treatment followed by semi-quantitative RT-PCR to detect expression of the *CRP4* gene and *MPK3*.

For heat stress, approximately one week-old seedlings were transferred into liquid MS medium and incubated at 4°C for 24 hr, and different batches were then incubated for 4 hrs, 8 hrs, and 24 hrs, respectively, at 37°C. Stressed seedlings were allowed to recover at 21°C for 3 days, and then used for semi-quantitative RT-PCR to detect expression of the *CRP4* gene and *Onsen* retrotransposon [19].

### **Accession numbers**

Accession numbers for the single *SAT5* monomers containing *CRP* genes or pseudogenes shown in Table 1 are as follows: chr2 *SAT5* fragment containing At2g18042 pseudogene [EMBL:HE610172]; chromosome 3 *SAT5* fragment containing At3g42565 gene [EMBL:HE610173]; chromosome 4 *SAT5* fragment containing At4g09545 gene [EMBL:HE610174]; chromosome 5 *SAT5* fragment containing At5g60978 pseudogene [EMBL:HE610175]. The small RNA sequence data are available from NCBI’s Gene Expression Omnibus (GEO) and are accessible via GEO Series accession number GSE34207.

## **Abbreviations**

AGI: Arabidopsis Genome Initiative; ATSAT5 (SAT5): Arabidopsis thaliana satellite sequence on chromosome 5; CMT3: Chromomethylase 3; CRP: Cysteine-rich peptide; CRP4: At4g09545; CRP5a: At5g42895; CRP5b: At5g44495; DCL3: Dicer-like3; DDM1: Decrease in dna methylation1; DMS4: Defective in meristem silencing 4; DRM: Domains rearranged methyltransferase; ECA1: Early culture abundant; EGFP: Enhanced GFP; GFP: Green fluorescent protein; GUS: ß-glucuronidase; LAM: Laser-assisted microdissection; MSAP: Methylation-sensitive amplification polymorphism; MET1: Methyltransferase 1; NRPD1: Largest subunit of nuclear RNA polymerase IV (Pol IV); NRPE1: Largest subunit of nuclear RNA polymerase V (Pol V); RDR2: RNA-DEPENDENT RNA POLYMERASE 2; RDR6: RNA-DEPENDENT RNA POLYMERASE 6; SAT5: Short name for ATSAT5; TAIR: The Arabidopsis Information Resource.

## **Authors’ contributions**

WY, AT, AM and MM designed the study; WY, AT, MS, LD SS, BM generated and interpreted data; MWS and UG helped with the LAM studies; WY, AT, LD, UG, MS, MS, SS, BM, AM, MM interpreted data; WY, AT, LD, MS, UG, SS, BM and MM prepared the paper. All authors read and approved the final manuscript.

## Supplementary Material

Additional file 1 **Figure S1.** Differentially accumulating fragments in MSAP analysis. Click here for file

Additional file 2 **Figure S2.** Sequence alignments. Click here for file

Additional file 3 **Table S1.** Methylation status of members of the CRP3600 subgroup. Click here for file

Additional file 4 **Table S3.** Primers. Click here for file
