# Supplementary material for: Atypical DNA methylation of genes encoding cysteine-rich peptides in Arabidopsis thaliana
Source: BMC Plant Biol. 2012 Apr 19;12:51. doi: 10.1186/1471-2229-12-51 (PMC3422182; doi:10.1186/1471-2229-12-51)
Supplement: Additional file 1 — Figure S1. Differentially accumulating fragments in MSAP analysis. [file 1471-2229-12-51-S1.pdf]

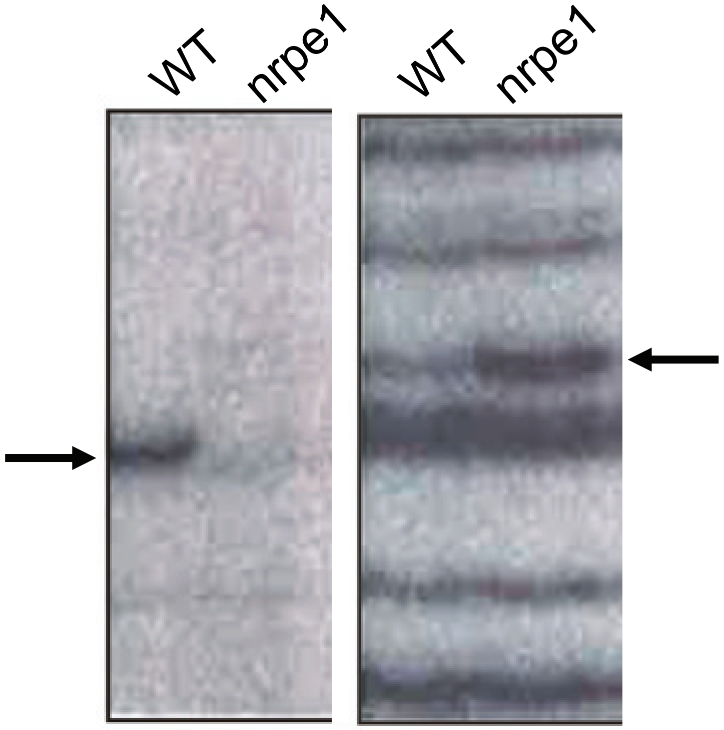

Supplementary Figure 1. Differentially accumulating fragments in MSAP analysis
Polymorphic bands between wild-type (WT) and *nrpe1* mutant seedlings on MSAP gels (arrows). These correspond to the SAT5 monomers on chromosomes 2 and 5 respectively, containing the pseudogenes At2g18042 and At5g60978 (Table 1).
