# Supplementary material for: Atypical DNA methylation of genes encoding cysteine-rich peptides in Arabidopsis thaliana
Source: BMC Plant Biol. 2012 Apr 19;12:51. doi: 10.1186/1471-2229-12-51 (PMC3422182; doi:10.1186/1471-2229-12-51)
Supplement: Additional file 2 — Figure S2. Sequence alignments. [file 1471-2229-12-51-S2.pdf]

Supplementary Figure 2. Sequence alignment

Section 1

		1 ... 10 ... 20 ... 30 ... 40 ... 50
	(1)	
ATSAT5Satellite	(1)	TTATGTTCATTGTTTTCATGCCAGATCCGAACTGCTCTTCCCAACCGTTT
chr.2_SAT5	(1)	--
chr.3_SAT5	(1)	TTATGTACAATGTTTTCATGCCCGATCCGAACTGCTCTGTCCAACCGTAT
Chr.4_SAT5	(1)	--
Chr.5_SAT5	(1)	--
AT2G18042	(1)	--
AT3G42565	(1)	--
AT4G09545	(1)	--
AT5G60978	(1)	--
Consensus	(1)	TTATGTTCATTGTTTTCATGCCCGATCCGAACTGCTCTTTCCAACCGTTT

Section 2

		51 ... 60 ... 70 ... 80 ... 90 ... 100
	(51)	
ATSAT5Satellite	(51)	AACCCGCTATCCAGAAACTCTTCTCCTCTAAGTTTAGTGTTAACAAGTCA
chr.2_SAT5	(1)	--
chr.3_SAT5	(51)	AATCCGCATTCTACAAACTCCTCTCGTCTAAGTTTAGTGTTAACAAGTCA
Chr.4_SAT5	(1)	--ACAAATCA
Chr.5_SAT5	(1)	--
AT2G18042	(1)	--
AT3G42565	(1)	--
AT4G09545	(1)	--
AT5G60978	(1)	--
Consensus	(51)	AATCCGCTTTCTAGAAACTCTTCTCGTCTAAGTTTAGTGTTAACAAGTCA

Section 3

		101 ... 110 ... 120 ... 130 ... 140 ... 150
	(101)	
ATSAT5Satellite	(101)	TGGAGAACCTCATTGTATAGAAGTTTTACAAACCAAACCTTTAGATTGAT
chr.2_SAT5	(1)	---GAT
chr.3_SAT5	(101)	TGGAGAACCCCATTGTATAGAAGTTTTACAAACCAAACCTTTAGATTGAT
Chr.4_SAT5	(9)	TGGAGAACC-CATTGTATAAAAATTTTACAAACCAAACCTTTAGGTCAAT
Chr.5_SAT5	(1)	--
AT2G18042	(1)	--
AT3G42565	(1)	--
AT4G09545	(1)	--
AT5G60978	(1)	--
Consensus	(101)	TGGAGAACCTCATTGTATAGAAGTTTTACAAACCAAACCTTTAGATTGAT

Section 4

		151 ... 160 ... 170 ... 180 ... 190 ... 200
	(151)	
ATSAT5Satellite	(151)	TCAAATAATTT-CACGAAGAAAATATTATAAATCGATTTATTTGTTTAAA
chr.2_SAT5	(4)	TCAAATAATTTTCTCTAGAAAATTCGAATAAATCGGTTTATTGGTTTAAA
chr.3_SAT5	(151)	TTAAATAATTTTCACGAAGAAAATATTATAAATCTATTTATTTGTTTAAA
Chr.4_SAT5	(58)	TCAAATAATTTTCTCTAAGAAAATATTATACATCGATTTGTTTGTTTGAA
Chr.5_SAT5	(1)	--
AT2G18042	(1)	--
AT3G42565	(1)	--
AT4G09545	(1)	--
AT5G60978	(1)	--
Consensus	(151)	TCAAATAATTTTCTCTAAGAAAATATTATAAATCGATTTATTTGTTTAAA

Supplementary Figure 2. Sequence alignment

Section 5

		201 210 220 230 240 250
	(201)	
ATSAT5Satellite	(200)	TTTGGAGATATAAACC-GATGATTGATTAATTATGTGAAGCC----ATTT
chr.2_SAT5	(54)	TTTTGAGATATATACT-GGTAACTGATTAATTATGTGAAGCC---GGTTA
chr.3_SAT5	(201)	TTTGGAGATATATACC-GATGAATGATTAATTATGTGAAGCC----GTTT
Chr.4_SAT5	(108)	TTTTGAGATATAAAACTGGTGATTGATTAATTATGTGAAGTCATACGTTA
Chr.5_SAT5	(1)	--
AT2G18042	(1)	--
AT3G42565	(1)	--
AT4G09545	(1)	--
AT5G60978	(1)	--
Consensus	(201)	TTTTGAGATATATACCTGGTGATTGATTAATTATGTGAAGCCATAGGTTT

Section 6

		251 260 270 280 290 300
	(251)	
ATSAT5Satellite	(245)	TACTTTTATTTTCTCT-TTGTAGTCAATATTTCTTAGTTAATGTTACGTG
chr.2_SAT5	(100)	CACTTTTATTG-CTCT-TTGTAGTCAATATTTCCAAGTTAATGTTACTTG
chr.3_SAT5	(246)	AACTTTGATTTTCTCT-TTGTAGTCAATATTTTTTAGTTAATGTTACTTG
Chr.4_SAT5	(158)	TACTTTTATTTTCTCTCTTTTAATCAATATTTCTTAGTTAATGTTGCTTC
Chr.5_SAT5	(1)	--
AT2G18042	(1)	--
AT3G42565	(1)	--
AT4G09545	(1)	--
AT5G60978	(1)	--
Consensus	(251)	TACTTTTATTTTCTCTCTTGTAGTCAATATTTCTTAGTTAATGTTACTTG

Section 7

		301 310 320 330 340 350
	(301)	
ATSAT5Satellite	(294)	TTTCACACATGGAGTTTCAACTATATT-AAATTTCTAAAACGGCTAATCA
chr.2_SAT5	(148)	TTTT---CTTGGAGTTTCAACTCTATTTATACTTATAAAACAACTATTGA
chr.3_SAT5	(295)	TTTCACACATGGAGTTTCAACTATATTTAAATTTATAAAACGGCTAATCA
Chr.4_SAT5	(208)	TTTTACACTTGGAGTTTCGTCTTTATTTTGATTTATAAAACTGTTAATCA
Chr.5_SAT5	(1)	--
AT2G18042	(1)	--
AT3G42565	(1)	--
AT4G09545	(1)	--
AT5G60978	(1)	--
Consensus	(301)	TTTTACACTTGGAGTTTCAACTATATTTAAATTTATAAAACGGCTAATCA

Section 8

		351 360 370 380 390 400
	(351)	
ATSAT5Satellite	(343)	AATACCTAATTAAAGTATTCAAAGATGTTAAATTAAA-AAAATTAATATG
chr.2_SAT5	(195)	AATTTCTAATTAAAGCATCCAAAGATGTTAAAGTAAG-ATAATTAATGTC
chr.3_SAT5	(345)	AATACCTAATTAAAGTATTCAAAGATGTTAAGTTAAAGAAAATCTATATG
Chr.4_SAT5	(258)	AATACCTAATTAAACCATTCAAAGATGTTA--TTAAA-ATAATTAATATC
Chr.5_SAT5	(1)	--
AT2G18042	(1)	--
AT3G42565	(1)	--
AT4G09545	(1)	--
AT5G60978	(1)	--
Consensus	(351)	AATACCTAATTAAAGTATTCAAAGATGTTAAATTAAAGATAATTAATATG

Supplementary Figure 2. Sequence alignment

Section 9

```
                 (401) 401      410       420       430       440      450
ATSAT5Satellite  (392) ACAGTTTCACATTTCAATC--CATATAAATAAGCAAGGATTTTATTGTTA
chr.2_SAT5       (244) ATAGTTTCACATTTCATTC--CATATAAATAATCAAAAGTCTTATTGTTG
chr.3_SAT5       (395) ATAGTTTCACATTTCAATATTCATATAAATAAGCAAGGATTTTATTGTTA
Chr.4_SAT5       (305) ACAGTTTTACATTTCATTC--CATATAAATAAGCAGGAATCTTATTGTTG
Chr.5_SAT5         (1) --------------------------------------------------
AT2G18042          (1) --------------------------------------------------
AT3G42565          (1) --------------------------------------------------
AT4G09545          (1) --------------------------------------------------
AT5G60978          (1) --------------------------------------------------
Consensus        (401) ATAGTTTCACATTTCATTCTTCATATAAATAAGCAAGGATTTTATTGTTG
```

Section 10

```
                 (451) 451      460       470       480       490      500
ATSAT5Satellite  (440) TTCATCAATCAAAAACAAACAAAGCATTAAGCATATAGTTG-CTATACTA
chr.2_SAT5       (292) TTCATCAATCAAAAGCAAATATAACCTTCAGCATATATTTGGCTATACTA
chr.3_SAT5       (445) TTCATCAATCAAAAAAAAACAAAGCATTAAGCATATAGTTG-CTATACTA
Chr.4_SAT5       (353) TTCATCAATCAAAAACAAACAGAGCATTAAGCATATATTTGGCTATACTA
Chr.5_SAT5         (1) --------------------------------------------------
AT2G18042          (1) --------------------------------------------------
AT3G42565          (1) --------------------------------------------------
AT4G09545          (1) --------------------------------------------------
AT5G60978          (1) --------------------------------------------------
Consensus        (451) TTCATCAATCAAAAACAAACAAAGCATTAAGCATATATTTGGCTATACTA
```

Section 11

```
                 (501) 501      510       520       530       540      550
ATSAT5Satellite  (489) AGACTTTTTA----CTCAATTTCTCATTATGTCTATCAAAAATGTGTTTT
chr.2_SAT5       (342) AGGTTCTTTT--TACTCAATTTCTCATTATGTCTATAAAAAATATGCTTT
chr.3_SAT5       (494) AGACTTTGTA----CTGAATTTCTCATTATGTCTATCAAAAATGTGTTTT
Chr.4_SAT5       (403) AGGCTTTTTTATTTCTCAGTTTCTCATTATGCCTATCAAAAATGTGTTTT
Chr.5_SAT5         (1) --------------------TTTTCATCATGGCTATCAAAAACTTGATTT
AT2G18042          (1) ----------------------------ATGTCTATAAAAAATATGCTTT
AT3G42565          (1) ----------------------------ATGTCTATCAAAAATGTGTTTT
AT4G09545          (1) ----------------------------ATGCCTATCAAAAATGTGTTTT
AT5G60978          (1) ----------------------------ATGGCTATCAAAAACTTGATTT
Consensus        (501) AGGCTTTTTTATTTCTCAATTTCTCATTATGTCTATCAAAAATGTGTTTT
```

Section 12

```
                 (551) 551      560       570       580       590      600
ATSAT5Satellite  (535) TACTTCTAGCGGTTCTATGTATCATAGTTTCTGTAAATGCTCAATTGCCT
chr.2_SAT5       (390) CACTCCTAGCGGTTCTATGCATCATCGTTTCTGTAAATGCTTAGATGCCT
chr.3_SAT5       (540) CCCTTCTATATGTTCTATGTATCATAGTTTCTGTAAATGCTCAATTGCCT
Chr.4_SAT5       (453) CACTCTTAGTGGTTCTATGCATCATAGTTTCTGTAAATGCTCAGTTACCT
Chr.5_SAT5        (31) TTATTATAGCGGTTATGTGCGTTTTTGTCTCCGTAAATGCTCAACTACCA
AT2G18042         (23) CACTCCTAGCGGTTCTATGCATCATCGTTTCTGTAAATGCTTAGATGCCT
AT3G42565         (23) CCCTTCTATATGTTCTATGTATCATAGTTTCTGTAAATGCTCAATTGCCT
AT4G09545         (23) CACTCTTAGTGGTTCTATGCATCATAGTTTCTGTAAATGCTCAGTTACCT
AT5G60978         (23) TTATTATAGCGGTTATGTGCGTTTTTGTCTCCGTAAATGCTCAACTACCA
Consensus        (551) CACTTCTAGCGGTTCTATGCATCATAGTTTCTGTAAATGCTCAATTGCCT
```

```
                                                                          Section 13
                    (601) 601      610       620       630       640        650
ATSAT5Satellite     (585) CAGTTTCCGGCTCAACTTCCTTTTCTGTTTCCGTTCCAACTGATTCCTGG
chr.2_SAT5          (440) CAGTTTCCGGCTCCATTTCCTTTTTCATTTCTATTCGAACCGATTCCTGG
chr.3_SAT5          (590) CAGTTTCCAGCTCAACTTCCTTTTCCGTTTCCGTTCCAACTGATTTCTGG
Chr.4_SAT5          (503) CAGTTTCCGGCTCCGTTTCTTTTTCCATTTTCGTTTCAACCGATTCCTGG
Chr.5_SAT5           (81) CA------------ATTTCCTTTTCCATTTTCATTCTAACCGATTTCTGG
AT2G18042            (73) CAGTTTCCGGCTCCATTTCCTTTTTCATTTCTATTCGAACCGATTCCTGG
AT3G42565            (73) CAGTTTCCAGCTCAACTTCCTTTTCCGTTTCCGTTCCAACTGATTTCTGG
AT4G09545            (73) CAGTTTCCGGCTCCGTTTCTTTTTCCATTTTCGTTTCAACCGATTCCTGG
AT5G60978            (73) CA------------ATTTCCTTTTCCATTTTCATTCTAACCGATTTCTGG
Consensus           (601) CAGTTTCCGGCTCCATTTCCTTTTCCATTTCCGTTCCAACCGATTCCTGG

                                                                          Section 14
                    (651) 651      660       670       680       690        700
ATSAT5Satellite     (635) A---------TTACCTGATATAACAAAATGTTTCTCATCCGTGATGGATA
chr.2_SAT5          (490) TATGTCAGGATTACCTGACATAACAAAATGTTGGTCATCCGTGATGGATA
chr.3_SAT5          (640) A---------TTACCTGATATAACAAAATGTTGGTCATCCGTGATGGATA
Chr.4_SAT5          (553) AATGCCAGGATTACCTGATATAACGAAATGTTGGTCATCCGTGATGGATA
Chr.5_SAT5          (119) TATGCCAGGATTACCTGATATTACAAAATGTTTGTCATCCGTGATGGATA
AT2G18042           (123) TATGTCAGGATTACCTGACATAACAAAATGTTGGTCATCCGTGATGGATA
AT3G42565           (123) A---------TTACCTGATATAACAAAATGTTGGTCATCCGTGATGGATA
AT4G09545           (123) AATGCCAGGATTACCTGATATAACGAAATGTTGGTCATCCGTGATGGATA
AT5G60978           (111) TATGCCAGGATTACCTGATATTACAAAATGTTTGTCATCCGTGATGGATA
Consensus           (651) AATGCCAGGATTACCTGATATAACAAAATGTTGGTCATCCGTGATGGATA

                                                                          Section 15
                    (701) 701      710       720       730       740        750
ATSAT5Satellite     (676) TTCCTGGATGCATTGCAGAGATTTCTCAATCCATTTTCACTGGAAAGTTC
chr.2_SAT5          (540) TTCCTGGATGCATTGTAGAGGTTTCTCAATCTATTTTCACTGGAAAATTC
chr.3_SAT5          (681) TTCCTGGATGCATTGCAGAGATTTCTCAATCCATTTTCACTGGAAAGTTC
Chr.4_SAT5          (603) TTCCTGAATGCATTGCAGAGATTTCTCAATCCATTTTTATTGGCAAATTC
Chr.5_SAT5          (169) TTCCGGGATGCATTGCAGAGATTTCTCAATCTATTTTCACAAGAAAATTT
AT2G18042           (173) TTCCTGGATGCATTGTAGAGGTTTCTCAATCTATTTTCACTGGAAAATTC
AT3G42565           (164) TTCCTGGATGCATTGCAGAGATTTCTCAATCCATTTTCACTGGAAAGTTC
AT4G09545           (173) TTCCTGAATGCATTGCAGAGATTTCTCAATCCATTTTTATTGGCAAATTC
AT5G60978           (161) TTCCGGGATGCATTGCAGAGATTTCTCAATCTATTTTCACAAGAAAATTT
Consensus           (701) TTCCTGGATGCATTGCAGAGATTTCTCAATCCATTTTCACTGGAAAATTC

                                                                          Section 16
                    (751) 751      760       770       780       790        800
ATSAT5Satellite     (726) GGTAATTTAGGTCCGGCTTGTTGCAAAGCATTTTTGGACGCCGA---TAA
chr.2_SAT5          (590) GGCAATTTAGGTCCATCTTGTTGTAAAGCATTGTTGGACGCTGAAGTCAA
chr.3_SAT5          (731) GGTAATTTAGGTCCGGCTTGTTGCAAAGCATTTTTGGATGCCAAAGTTAA
Chr.4_SAT5          (653) AGTAATTTAGGTTCTGCTTGTTGCAAAGCATTTTTGGACGCTGAAGCCAA
Chr.5_SAT5          (219) GGTAACTTAGGTCCGGCTTGTTGCAAAGCATTTTTGGATGTCGAATCCAA
AT2G18042           (223) GGCAATTTAGGTCCATCTTGTTGTAAAGCATTGTTGGACGCTGAAGTCAA
AT3G42565           (214) GGTAATTTAGGTCCGGCTTGTTGCAAAGCATTTTTGGATGCCAAAGTTAA
AT4G09545           (223) AGTAATTTAGGTTCTGCTTGTTGCAAAGCATTTTTGGACGCTGAAGCCAA
AT5G60978           (211) GGTAACTTAGGTCCGGCTTGTTGCAAAGCATTTTTGGATGTCGAATCCAA
Consensus           (751) GGTAATTTAGGTCCGGCTTGTTGCAAAGCATTTTTGGACGCCGAAGTCAA
```

Supplementary Figure 2. Sequence alignment

```
                                                                  Section 17
                  (801) 801      810       820       830       840      850
ATSAT5Satellite   (773) TTGCATACCGAAAATTCCATTCATTCCATTTTTTCCTCCGATGCTAAAGG
chr.2_SAT5        (640) TTCCATGCCGAAAATTCCATTCATTCTGTTTTTCCCTCCGATGCTAAAGG
chr.3_SAT5        (781) TTGCATACCGAAAATTCCATTCATTCCGTTATTCCCTCCGATGCTAAAGG
Chr.4_SAT5        (703) CTGCATACCAAAAATTCCACTTATTCCGCTTTTCCCTCCGATGCTAAAAG
Chr.5_SAT5        (269) TTGCATGCCAAAAATTCCATTCATTCCGTTATTTCCGCCGATGCTAAAAG
AT2G18042         (273) TTCCATGCCGAAAATTCCATTCATTCTGTTTTTCCCTCCGATGCTAAAGG
AT3G42565         (264) TTGCATACCGAAAATTCCATTCATTCCGTTATTCCCTCCGATGCTAAAGG
AT4G09545         (273) CTGCATACCAAAAATTCCACTTATTCCGCTTTTCCCTCCGATGCTAAAAG
AT5G60978         (261) TTGCATGCCAAAAATTCCATTCATTCCGTTATTTCCGCCGATGCTAAAAG
Consensus         (801) TTGCATACCGAAAATTCCATTCATTCCGTTTTTCCCTCCGATGCTAAAGG
                                                                  Section 18
                  (851) 851      860       870       880       890      900
ATSAT5Satellite   (823) AACAATGTTCAAGAGTTGCCGGTGCAACTCCTCCCATACCAAAATAGTGT
chr.2_SAT5        (690) AACAATGCTCAAGAATTGTGGGTGCAACTCCTCCTATACCAAAATAGAGT
chr.3_SAT5        (831) AACAATGTTCAAGAGTTGCCGGTGCAACTCCTCCCATACCAAAATAGAGT
Chr.4_SAT5        (753) AACAATGTTCAAGAATTGCCGGTGTAACTCCTCCTACACCGAAATAGAGT
Chr.5_SAT5        (319) AACAATGTTCAAGAATTGCCGGTGCTACTCCTCCTACACCAAAATAGAGT
AT2G18042         (323) AACAATGCTCAAGAATTGTGGGTGCAACTCCTCCTATACCAAAATAG---
AT3G42565         (314) AACAATGTTCAAGAGTTGCCGGTGCAACTCCTCCCATACCAAAATAG---
AT4G09545         (323) AACAATGTTCAAGAATTGCCGGTGTAACTCCTCCTACACCGAAATAG---
AT5G60978         (311) AACAATGTTCAAGAATTGCCGGTGCTACTCCTCCTACACCAAAATAG---
Consensus         (851) AACAATGTTCAAGAATTGCCGGTGCAACTCCTCCTATACCAAAATAGAGT
                                                                  Section 19
                  (901) 901      910       920       930       940      950
ATSAT5Satellite   (873) TTTATCTTATT---------TTTAAAATAGATAACTTTTT------CAAC
chr.2_SAT5        (740) TTTAGCTTAGT--T------TTAAAAATCTATGATTTTTTTCTTTTCAAC
chr.3_SAT5        (881) TTTAACTTATT---------TTAAAAATAGATAGCTTTTT------CAAC
Chr.4_SAT5        (803) TTTAAATTATTATT------TTTTAAATAGATAATTTTTTT---TCCAAT
Chr.5_SAT5        (369) CTTAGTTTATTTTTAAAACATATAAAATATGAATTTTTTGTCTTTTCAAC
AT2G18042         (370) --------------------------------------------------
AT3G42565         (361) --------------------------------------------------
AT4G09545         (370) --------------------------------------------------
AT5G60978         (358) --------------------------------------------------
Consensus         (901) TTTAGCTTATTTTTAAAACATTTAAAATAGATAATTTTTTTCTTTTCAAC
                                                                  Section 20
                  (951) 951      960       970       980       990     1000
ATSAT5Satellite   (908) TTATTTTTTT------AAAATTTT-AGCTTACGTTACATGTTATAATAAT
chr.2_SAT5        (782) TTATTTATT-------GAAATTGT-AGCTTACTCTACATGTTATACTCCA
chr.3_SAT5        (916) TTATGTTTT-------AAAATTTT-AGCTTTAGT-ACATGTTATAATAAT
Chr.4_SAT5        (844) TTATTTCTT-------ACAATTTT-AGCTTACGCTACATGTTATACTCCA
Chr.5_SAT5        (419) TTCTTTCTTAAATTTTATAATTCTCAGATTACTCAACATGTTTTACTTAA
AT2G18042         (370) --------------------------------------------------
AT3G42565         (361) --------------------------------------------------
AT4G09545         (370) --------------------------------------------------
AT5G60978         (358) --------------------------------------------------
Consensus         (951) TTATTTTTTTAATTTTAAAATTTTCAGCTTACGCTACATGTTATACTCAA
```

Supplementary Figure 2. Sequence alignment

```
                                                                    Section 21
                 (1001) 1001     1010      1020      1030      1040      1050
ATSAT5Satellite   (951) AATTAAAAAAAGAAGAAGATATAGTAATAACATTTGAGAAGTTGCATTTG
chr.2_SAT5        (824) AAAAGAAAAAA-ACGATGATACAATAATAACAT------AGTTGTATTTG
chr.3_SAT5        (957) AATAAAAAAA----GATTATATAGTAATAACATTTGAGAATTTGCATTTG
Chr.4_SAT5        (886) AAAAAATATC------ATATAAAGTAAAAACATTTGAGAAGTTGTATTTG
Chr.5_SAT5        (469) AAAAAAACGA------TGATACATTAATAACATTTGAGAAGTCGTATTTG
AT2G18042         (370) --------------------------------------------------
AT3G42565         (361) --------------------------------------------------
AT4G09545         (370) --------------------------------------------------
AT5G60978         (358) --------------------------------------------------
Consensus        (1001) AAAAAAAAAAAGACGATGATATAGTAATAACATTTGAGAAGTTGTATTTG
                                                                    Section 22
                 (1051) 1051     1060      1070      1080      1090      1100
ATSAT5Satellite  (1001) GGTTCCATGTTTATACATTCTTCACTTCTAATGAATAACACCTTGAAACA
chr.2_SAT5        (867) GGTTCAATACTTACTCATTTTTCAATTATAATGAATAACACCTTGAAACA
chr.3_SAT5       (1003) GGTTCAATGTCTACTCATTCTTTAATTCTAATGAATAACACCTTGAAACA
Chr.4_SAT5        (930) GGTTCAATATTTACTCATTCTTCAATTCTAATGAATAACAGCTTGAAACA
Chr.5_SAT5        (513) TGTTTAATTTTTAGTCGTTCTTTATTTCTAATGAATAATACCTTCAAACA
AT2G18042         (370) --------------------------------------------------
AT3G42565         (361) --------------------------------------------------
AT4G09545         (370) --------------------------------------------------
AT5G60978         (358) --------------------------------------------------
Consensus        (1051) GGTTCAATGTTTACTCATTCTTCAATTCTAATGAATAACACCTTGAAACA
                                                                    Section 23
                 (1101) 1101     1110      1120      1130      1140      1150
ATSAT5Satellite  (1051) TCGAGTCCATAATTGAATTTAGTCTTACAATGTTACAGACCTTCAAATTG
chr.2_SAT5        (917) TTAACTCCGTAATT-AATTTAGTCTTGCAAT-------------------
chr.3_SAT5       (1053) TCGAGCCCATAAT---G-------TTACAATGTTACAGAATTTCAAATTG
Chr.4_SAT5        (980) TCGATTCTATAATT-AACTTTGTCTTACAATGTT----------------
Chr.5_SAT5        (563) TCGAGTAGATAATT-AATTTAATATTGCAATATTTCAGAATTT---GTTG
AT2G18042         (370) --------------------------------------------------
AT3G42565         (361) --------------------------------------------------
AT4G09545         (370) --------------------------------------------------
AT5G60978         (358) --------------------------------------------------
Consensus        (1101) TCGAGTCCATAATTGAATTTAGTCTTACAATGTTACAGAATTTCAAATTG
                                                                    Section 24
                 (1151) 1151     1160      1170      1180      1190      1200
ATSAT5Satellite  (1101) TTTCCATAAATCTTTGAAATATGTCATA-----ACACAAAATCTGAGAGT
chr.2_SAT5        (947) --------------------------------------------------
chr.3_SAT5       (1093) TTTTCATAAATCTTTGAAATATGTCATA-----ACACAAAATCTGAGAAT
Chr.4_SAT5       (1013) --------------------------------------------------
Chr.5_SAT5        (609) TTTTCATAAATCTTTTAGATACGTCGTATAGTAACACAAAATCTGTCAAC
AT2G18042         (370) --------------------------------------------------
AT3G42565         (361) --------------------------------------------------
AT4G09545         (370) --------------------------------------------------
AT5G60978         (358) --------------------------------------------------
Consensus        (1151) TTTTCATAAATCTTTGAAATATGTCATATAGTAACACAAAATCTGAGAAT
```

Supplementary Figure 2. Sequence alignment

```
                                                                     Section 25
                 (1201) 1201     1210      1220      1230      1240      1250
ATSAT5Satellite (1146) GGTTTATAATTTTATTAGATAAGACTCGTCCAAATATGCTGGTGTGAGAA
     chr.2_SAT5  (947) --------------------------------------------------
     chr.3_SAT5 (1138) GGTTTATAATTTTATTAGATAAGACTCGTCTAA-TAGGCGGGTGTGAGAA
     Chr.4_SAT5 (1013) --------------------------------------------------
     Chr.5_SAT5  (659) GGTTTATAACTTT-TTATATACGAC-------------------------
      AT2G18042  (370) --------------------------------------------------
      AT3G42565  (361) --------------------------------------------------
      AT4G09545  (370) --------------------------------------------------
      AT5G60978  (358) --------------------------------------------------
      Consensus (1201) GGTTTATAATTTTATTAGATAAGACTCGTCTAAATATGCTGGTGTGAGAA
                                                                     Section 26
                 (1251) 1251     1260      1270      1280      1290      1300
ATSAT5Satellite (1196) ATGTTGTGATGATATAAAAATTCTAAATAGTAATCGGTATTTTTCAAAAG
     chr.2_SAT5  (947) --------------------------------------------------
     chr.3_SAT5 (1187) ATGTTGTGATGATATAAAAATTCTAAATAGTAATCTGTATTTT-CAAAAG
     Chr.4_SAT5 (1013) --------------------------------------------------
     Chr.5_SAT5  (683) --------------------------------------------------
      AT2G18042  (370) --------------------------------------------------
      AT3G42565  (361) --------------------------------------------------
      AT4G09545  (370) --------------------------------------------------
      AT5G60978  (358) --------------------------------------------------
      Consensus (1251) ATGTTGTGATGATATAAAAATTCTAAATAGTAATCTGTATTTTTCAAAAG
                                                                     Section 27
                 (1301) 1301     1310      1320      1330      1340      1350
ATSAT5Satellite (1246) AATATTTCAATTATATACATTCCATATACCATGCCTTTACTAATTATATA
     chr.2_SAT5  (947) --------------------------------------------------
     chr.3_SAT5 (1236) AATATTTCAATTATACACATTCTATATACCATGCATTTTCTAATTATATA
     Chr.4_SAT5 (1013) --------------------------------------------------
     Chr.5_SAT5  (683) --------------------------------------------------
      AT2G18042  (370) --------------------------------------------------
      AT3G42565  (361) --------------------------------------------------
      AT4G09545  (370) --------------------------------------------------
      AT5G60978  (358) --------------------------------------------------
      Consensus (1301) AATATTTCAATTATATACATTCTATATACCATGCCTTTTCTAATTATATA
                                                                     Section 28
                 (1351) 1351     1360      1370      1380      1390      1400
ATSAT5Satellite (1296) TAATACGGATTTCTCAAAGTATAAAAGAAAGATAAGACAAGGAAAACAT
     chr.2_SAT5  (947) --------------------------------------------------
     chr.3_SAT5 (1286) TTATACGGAATCCTCAAAGCATAACACAAAGATAAGACAAGGAAAACAT
     Chr.4_SAT5 (1013) --------------------------------------------------
     Chr.5_SAT5  (683) --------------------------------------------------
      AT2G18042  (370) --------------------------------------------------
      AT3G42565  (361) --------------------------------------------------
      AT4G09545  (370) --------------------------------------------------
      AT5G60978  (358) --------------------------------------------------
      Consensus (1351) TTATACGGGATTTCTCAAAGTATAACAGAAAGATAAGACAAGGAAAACAT
```

Supplementary Figure 2. Sequence alignment

```
                                                                    Section 29
           (1401) 1401     1410      1420      1430      1440      1450
ATSAT5Satellite (1346) ACAACATCTATAAAA-GACGCACCAGACAGAGCCTCTCACTGAATTTTGT
     chr.2_SAT5  (947) --------------------------------------------------
     chr.3_SAT5 (1336) ACAACAGCTTTAAAAAGACGCACCAAACAGAGCCTCTCACTGAATTTTGT
     Chr.4_SAT5 (1013) --------------------------------------------------
     Chr.5_SAT5  (683) --------------------------------------------------
       AT2G18042 (370) --------------------------------------------------
       AT3G42565 (361) --------------------------------------------------
       AT4G09545 (370) --------------------------------------------------
       AT5G60978 (358) --------------------------------------------------
      Consensus (1401) ACAACATCTTTAAAAAGACGCACCAGACAGAGCCTCTCACTGAATTTTGT
                                                                    Section 30
           (1451) 1451     1460      1470      1480      1490      1500
ATSAT5Satellite (1395) GGTAAACGAATAGAACTCTGTAATTGAGAAAAAAATAAATGAAATAATAT
     chr.2_SAT5  (947) --------------------------------------------------
     chr.3_SAT5 (1386) GGTAAACGAATTGAACTCCGTAATTGAGAAAAAATGAAATGAAATAAAAT
     Chr.4_SAT5 (1013) --------------------------------------------------
     Chr.5_SAT5  (683) --------------------------------------------------
       AT2G18042 (370) --------------------------------------------------
       AT3G42565 (361) --------------------------------------------------
       AT4G09545 (370) --------------------------------------------------
       AT5G60978 (358) --------------------------------------------------
      Consensus (1451) GGTAAACGAATTGAACTCTGTAATTGAGAAAAAATTAAATGAAATAATAT
                                                                    Section 31
           (1501) 1501     1510      1520      1530      1540      1550
ATSAT5Satellite (1445) TAAGAAATAAAAAGAGAGACATATTTAAGTTACAAAATTAGAAAATATTA
     chr.2_SAT5  (947) --------------------------------------------------
     chr.3_SAT5 (1436) TAAGAAATAAAAGGAGAGACATATTTAAGTTACAAAATTAGAAAATATTA
     Chr.4_SAT5 (1013) --------------------------------------------------
     Chr.5_SAT5  (683) --------------------------------------------------
       AT2G18042 (370) --------------------------------------------------
       AT3G42565 (361) --------------------------------------------------
       AT4G09545 (370) --------------------------------------------------
       AT5G60978 (358) --------------------------------------------------
      Consensus (1501) TAAGAAATAAAAGGAGAGACATATTTAAGTTACAAAATTAGAAAATATTA
                                                                    Section 32
           (1551) 1551     1560      1570      1580      1590      1600
ATSAT5Satellite (1495) AATCACGTATTAATATTTTTCTAAAACAAAATAGGGAACTTTTACTTCTG
     chr.2_SAT5  (947) --------------------------------------------------
     chr.3_SAT5 (1486) AATCAAGTATTAATATTTTTCTAAAACAAAATAGGGAACTTTTACTTCTG
     Chr.4_SAT5 (1013) --------------------------------------------------
     Chr.5_SAT5  (683) --------------------------------------------------
       AT2G18042 (370) --------------------------------------------------
       AT3G42565 (361) --------------------------------------------------
       AT4G09545 (370) --------------------------------------------------
       AT5G60978 (358) --------------------------------------------------
      Consensus (1551) AATCACGTATTAATATTTTTCTAAAACAAAATAGGGAACTTTTACTTCTG
```

Supplementary Figure 2. Sequence alignment

```
                                                                        Section 33
                      (1601) 1601     1610      1620      1630      1640      1650
ATSAT5Satellite (1545) TTTTGAATATATAAACGATTTTTAATAGAATTTGTTCAATATTTTAATAT
     chr.2_SAT5  (947) --------------------------------------------------
     chr.3_SAT5 (1536) TTTTGAATATATAAACGACATTTAATAGAATTTGTTCAATAGTTTAATAT
     Chr.4_SAT5 (1013) --------------------------------------------------
     Chr.5_SAT5  (683) --------------------------------------------------
      AT2G18042  (370) --------------------------------------------------
      AT3G42565  (361) --------------------------------------------------
      AT4G09545  (370) --------------------------------------------------
      AT5G60978  (358) --------------------------------------------------
      Consensus (1601) TTTTGAATATATAAACGATTTTTAATAGAATTTGTTCAATATTTTAATAT
                                                                        Section 34
                      (1651) 1651     1660      1670      1680      1690      1700
ATSAT5Satellite (1595) AGTAAATAAAAAATTTGTATATATGAACTA--TTTTTTTTACTTGTGTAT
     chr.2_SAT5  (947) --------------------------------------------------
     chr.3_SAT5 (1586) ATTAAATAAACAATTTGAATATATGAACTAACTTTTTTTTACTTATGTAT
     Chr.4_SAT5 (1013) --------------------------------------------------
     Chr.5_SAT5  (683) --------------------------------------------------
      AT2G18042  (370) --------------------------------------------------
      AT3G42565  (361) --------------------------------------------------
      AT4G09545  (370) --------------------------------------------------
      AT5G60978  (358) --------------------------------------------------
      Consensus (1651) ATTAAATAAACAATTTGTATATATGAACTAACTTTTTTTTACTTGTGTAT
                                                                        Section 35
                      (1701) 1701     1710      1720      1730      1740      1750
ATSAT5Satellite (1643) ATATCCGATGGGGTTATGAAATTTTTCTATATTTTGTTTTTTAAAAATAT
     chr.2_SAT5  (947) --------------------------------------------------
     chr.3_SAT5 (1636) ATATCCGATGGGGTTATGAAATTTTTCTAT-TTTTGTTTT---AAAATAT
     Chr.4_SAT5 (1013) --------------------------------------------------
     Chr.5_SAT5  (683) --------------------------------------------------
      AT2G18042  (370) --------------------------------------------------
      AT3G42565  (361) --------------------------------------------------
      AT4G09545  (370) --------------------------------------------------
      AT5G60978  (358) --------------------------------------------------
      Consensus (1701) ATATCCGATGGGGTTATGAAATTTTTCTATATTTTGTTTTTTAAAAATAT
                                                                        Section 36
                      (1751) 1751     1760      1770      1780      1790      1800
ATSAT5Satellite (1693) AGGGTTTTGTGCAAAACACCCATCTAACTCATCTAGTTTTGCAATTTTAC
     chr.2_SAT5  (947) --------------------------------------------------
     chr.3_SAT5 (1682) AGGATTTTGTGCAAAACACCCATCTAACTCATCCTATTTTGCAATTTTAC
     Chr.4_SAT5 (1013) --------------------------------------------------
     Chr.5_SAT5  (683) --------------------------------------------------
      AT2G18042  (370) --------------------------------------------------
      AT3G42565  (361) --------------------------------------------------
      AT4G09545  (370) --------------------------------------------------
      AT5G60978  (358) --------------------------------------------------
      Consensus (1751) AGGGTTTTGTGCAAAACACCCATCTAACTCATCTTGTTTTGCAATTTTAC
```

Supplementary Figure 2. Sequence alignment

```
                                                                     Section 37
             (1801) 1801     1810      1820      1830      1840      1850
ATSAT5Satellite (1743) CCTATATCTCTAACAAACACATATTAATCTTATGAAGTCTAAAATAATAC
     chr.2_SAT5  (947) --------------------------------------------------
     chr.3_SAT5 (1732) CTTCTATCTCTAACAAATGCATATTAATCTTATGAAGACTAAAAGAATAC
     Chr.4_SAT5 (1013) --------------------------------------------------
     Chr.5_SAT5  (683) --------------------------------------------------
      AT2G18042  (370) --------------------------------------------------
      AT3G42565  (361) --------------------------------------------------
      AT4G09545  (370) --------------------------------------------------
      AT5G60978  (358) --------------------------------------------------
      Consensus (1801) CTTCTATCTCTAACAAATGCATATTAATCTTATGAAGTCTAAAATAATAC
                                                                     Section 38
             (1851) 1851     1860      1870      1880      1890      1900
ATSAT5Satellite (1793) GCAATATGACCCTTATACGTATATTGTAAACTTTTTTACACTCCCATAGA
     chr.2_SAT5  (947) --------------------------------------------------
     chr.3_SAT5 (1782) GTAATATGACCCTTATATGTATATAGTAAATATTTTTACAATCACATACA
     Chr.4_SAT5 (1013) --------------------------------------------------
     Chr.5_SAT5  (683) --------------------------------------------------
      AT2G18042  (370) --------------------------------------------------
      AT3G42565  (361) --------------------------------------------------
      AT4G09545  (370) --------------------------------------------------
      AT5G60978  (358) --------------------------------------------------
      Consensus (1851) GTAATATGACCCTTATATGTATATTGTAAATTTTTTTACACTCCCATAGA
                                                                     Section 39
             (1901) 1901     1910      1920      1930      1940      1950
ATSAT5Satellite (1843) TTAGATTTTTAATATTTCGAAGGTCGATTTGAACTTTTCGTCGAGAAGGC
     chr.2_SAT5  (947) --------------------------------------------------
     chr.3_SAT5 (1832) TTAGATTTTTAAAATTTTGTTGGTAGAGTTGAAGTTTTCGTTGCGAGGGC
     Chr.4_SAT5 (1013) --------------------------------------------------
     Chr.5_SAT5  (683) --------------------------------------------------
      AT2G18042  (370) --------------------------------------------------
      AT3G42565  (361) --------------------------------------------------
      AT4G09545  (370) --------------------------------------------------
      AT5G60978  (358) --------------------------------------------------
      Consensus (1901) TTAGATTTTTAATATTTTGTTGGTCGATTTGAAGTTTTCGTTGCGAGGGC
                                                                     Section 40
             (1951) 1951     1960      1970      1980      1990      2000
ATSAT5Satellite (1893) TTCGATTAAACATATTAAATATCAATATCTTACAAAATATTGTCGTAGAA
     chr.2_SAT5  (947) --------------------------------------------------
     chr.3_SAT5 (1882) TTCAATTTAACATATTATATATCAATATTTTACAAACATTTATGGTAGCA
     Chr.4_SAT5 (1013) --------------------------------------------------
     Chr.5_SAT5  (683) --------------------------------------------------
      AT2G18042  (370) --------------------------------------------------
      AT3G42565  (361) --------------------------------------------------
      AT4G09545  (370) --------------------------------------------------
      AT5G60978  (358) --------------------------------------------------
      Consensus (1951) TTCGATTTAACATATTATATATCAATATTTTACAAACTTTTGTGGTAGCA
```

Supplementary Figure 2. Sequence alignment

Section 41

```
                   (2001) 2001     2010      2020      2030      2040      2050
ATSAT5Satellite (1943) AAA--TGACGAAAAGGGGTTACATTGCGTATTATTTTAGACTTCATCGGA
chr.2_SAT5       (947) --------------------------------------------------
chr.3_SAT5      (1932) AAAATTGACGAAGAAAGGTTGCAATGGGTATTATTTTAGACTTCGTAGGA
Chr.4_SAT5      (1013) --------------------------------------------------
Chr.5_SAT5       (683) --------------------------------------------------
AT2G18042        (370) --------------------------------------------------
AT3G42565        (361) --------------------------------------------------
AT4G09545        (370) --------------------------------------------------
AT5G60978        (358) --------------------------------------------------
Consensus       (2001) AAAATTGACGAAGAGGGGTTGCATTGGGTATTATTTTAGACTTCGTCGGA
```

Section 42

```
                   (2051) 2051     2060      2070      2080      2090      2100
ATSAT5Satellite (1991) TAATTATGCATTTTTTGGAGGTGGAGAATAAAGATGCAAAAATAGATGAG
chr.2_SAT5       (947) --------------------------------------------------
chr.3_SAT5      (1982) TAAT-ATGCAGTTGTTGGAGGTGGAGGATAAATATGCAAAAATAGATGAG
Chr.4_SAT5      (1013) --------------------------------------------------
Chr.5_SAT5       (683) --------------------------------------------------
AT2G18042        (370) --------------------------------------------------
AT3G42565        (361) --------------------------------------------------
AT4G09545        (370) --------------------------------------------------
AT5G60978        (358) --------------------------------------------------
Consensus       (2051) TAATTATGCATTTTTTGGAGGTGGAGGATAAATATGCAAAAATAGATGAG
```

Section 43

```
                   (2101) 2101     2110      2120      2130      2140      2150
ATSAT5Satellite (2041) TTAGAGGGTTTTAT-TTTGCACAAAACCCTAAAAATAACAAAATTTGATT
chr.2_SAT5       (947) --------------------------------------------------
chr.3_SAT5      (2031) TTAGAGAGTTTTTTCTTTGCACATAACCCTAAAAATAACGAAATT-GATT
Chr.4_SAT5      (1013) --------------------------------------------------
Chr.5_SAT5       (683) --------------------------------------------------
AT2G18042        (370) --------------------------------------------------
AT3G42565        (361) --------------------------------------------------
AT4G09545        (370) --------------------------------------------------
AT5G60978        (358) --------------------------------------------------
Consensus       (2101) TTAGAGGGTTTTTTCTTTGCACATAACCCTAAAAATAACGAAATTTGATT
```

Section 44

```
                   (2151) 2151     2160      2170      2180      2190      2200
ATSAT5Satellite (2090) TGATAAGGGTGGATATTTCACTAATAAATTTGACACGTGTCATGATCACG
chr.2_SAT5       (947) --------------------------------------------------
chr.3_SAT5      (2080) TGATATGTGTGGATAACTCATTAATAATTTTGACACGTGTCATGATCAGG
Chr.4_SAT5      (1013) --------------------------------------------------
Chr.5_SAT5       (683) --------------------------------------------------
AT2G18042        (370) --------------------------------------------------
AT3G42565        (361) --------------------------------------------------
AT4G09545        (370) --------------------------------------------------
AT5G60978        (358) --------------------------------------------------
Consensus       (2151) TGATATGTGTGGATATTTCATTAATAATTTTGACACGTGTCATGATCAGG
```

Supplementary Figure 2. Sequence alignment

```
                                                                      Section 45
                  (2201) 2201     2210      2220      2230      2240      2250
ATSAT5Satellite   (2140) TGAGTTAACAATTTTGAAAGCCCAACTTTATGTA----GATATTTGGCAC
chr.2_SAT5         (947) --------------------------------------------------
chr.3_SAT5        (2130) TGAGTTAACAATTTTGAAAACTCAACTTTGTATATTAAGATATTTGGCA-
Chr.4_SAT5        (1013) --------------------------------------------------
Chr.5_SAT5         (683) --------------------------------------------------
AT2G18042          (370) --------------------------------------------------
AT3G42565          (361) --------------------------------------------------
AT4G09545          (370) --------------------------------------------------
AT5G60978          (358) --------------------------------------------------
Consensus         (2201) TGAGTTAACAATTTTGAAAGCTCAACTTTGTGTATTAAGATATTTGGCAC
                                                                      Section 46
                  (2251) 2251       2261
ATSAT5Satellite   (2186) ATATATTGGAT
chr.2_SAT5         (947) -----------
chr.3_SAT5        (2179) -----------
Chr.4_SAT5        (1013) -----------
Chr.5_SAT5         (683) -----------
AT2G18042          (370) -----------
AT3G42565          (361) -----------
AT4G09545          (370) -----------
AT5G60978          (358) -----------
Consensus         (2251) ATATATTGGAT
```

Supplementary Figure 2. Sequence alignments

The 2196 bp SAT5 (ATSAT5) consensus sequence [1] is shown together with the four dispersed SAT5 monomers containing *CRP* genes and the actual coding sequences of the four *CRP* genes or pseudogenes embedded in them (Table 1). Shading indicates sequence identity. Yellow shading indicates *CRP* coding regions. The flanking regions are AT-rich compared to the *CRP* coding sequence (Supplementary Table 2). These AT-rich flanking regions are likely to provide transcriptional regulatory regions and indeed putative TATA boxes (TATAAA between 420 and 430 bp) and poly-adenylation sites (AATAA located between 1021 and 1030 bp) can be detected in these sequences. Alignments were made using Vector NTI Advance11 from Invitrogen. Accession numbers for the single SAT5 monomers containing *CRP* genes or pseudogenes are as follows: chr2 SAT5 fragment containing At2g18042 pseudogene [EMBL:HE610172]; chromosome 3 SAT5 fragment containing At3g42565 gene [EMBL:HE610173]; chromosome 4 SAT5 fragment containing At4g09545 gene [EMBL:HE610174]; chromosome 5 SAT5 fragment containing At5g60978 pseudogene [EMBL:HE610175].

[1] Genetic Information Research Institute: **ATSAT5: A satellite-like sequence from chromosome 5 – a consensus.** *Repbase Reports* 2006, **6**: 50.
