# Supplementary material for: Atypical DNA methylation of genes encoding cysteine-rich peptides in Arabidopsis thaliana
Source: BMC Plant Biol. 2012 Apr 19;12:51. doi: 10.1186/1471-2229-12-51 (PMC3422182; doi:10.1186/1471-2229-12-51)
Supplement: Additional file 3 — Table S1. Methylation status of members of the CRP3600 subgroup. [file 1471-2229-12-51-S3.pdf]

Supplementary Table 1. Methylation status of members of the CRP3600 subgroup

AGI	Intron Number	Pseudogene	Start Position	End Position	Methylation status	SAT5 embedded
At2g14378	0	F	6112313	6111951	None	No
At2g18042	0	T	7851529	7851897	Transposon	Yes
At2g21905	0	T	9346247	9346557	Transposon	No
At2g24205	0	F	10298498	10298842	CG and CHG	No
At2g27315	0	F	11697031	11696669	CG	No
At3g28985	0	T	10999264	10999576	Transposon	No
At3g42565	0	F	14694396	14694037	Transposon	Yes
At3g48675	0	F	18045177	18045536	None	No
At4g09545	0	F	6037566	6037934	Transposon	Yes
At4g35165	0	F	16735297	16735659	None	No
At5g17340	0	F	5716220	5715738	None	No
At5g36310	0	F	14342288	14341941	None	Yes
At5g36320	0	F	14344487	14344131	Transposon	Yes
At5g36330	0	T	14346684	14346328	Less	Yes
At5g36340	0	F	14348863	14348516	None	Yes
At5g36350	0	F	14351059	14350703	None	Yes
At5g36360	0	F	14353254	14352898	Less	Yes
At5g36370	0	F	14355451	14355095	None	Yes
At5g36380	0	F	14357648	14357292	None	Yes
At5g36390	0	F	14359843	14359487	None	Yes
At5g36400	0	F	14362038	14361682	None	Yes
At5g36410	0	F	14364233	14363877	None	Yes
At5g36420	0	F	14366428	14366072	None	Yes
At5g36430	0	F	14368623	14368267	None	Yes
At5g36440	0	F	14370819	14370463	None	Yes
At5g36450	0	F	14373016	14372660	None	Yes
At5g36460	0	F	14375212	14374856	Less	Yes
At5g36470	0	F	14377409	14377053	None	Yes
At5g36480	0	F	14379606	14379250	Less	Yes
At5g36490	0	F	14381803	14381447	None	Yes
At5g36500	0	F	14384000	14383644	None	Yes
At5g36510	0	T	14386198	14385842	Less	Yes
At5g36520	0	F	14388400	14388044	None	Yes
At5g36530	0	T	14390594	14390238	Less	Yes
At5g36540	0	F	14392787	14392431	None	Yes
At5g36550	0	F	14394980	14394624	None	Yes
At5g36657	0	F	14401406	14401107	CG and CHG	Yes
At5g36658	0	F	14403658	14403302	CG and CHG	Yes
At5g36659	0	F	14405851	14405552	CG	Yes
At5g36661	0	F	14408015	14407659	None	Yes
At5g36662	0	F	14410205	14409849	None	Yes
At5g36738	0	F	14467314	14466958	None	Yes
At5g36739	0	F	14469504	14469148	None	Yes
At5g42895	0	F	17214023	17214391	Transposon	No
At5g44495	0	F	17943937	17943578	Transposon	No

At5g44555	0	T	17962036	17962394	Transposon	No
At5g48210	0	F	19565685	19565996	None	No
At5g51105	0	F	20794858	20795229	None	No
At5g52965	0	F	21496067	21496429	None	No
At5g52975	0	F	21498740	21499105	None	No
At5g53742	0	F	21830897	21831244	Less	No
At5g53905	2	F	21906480	21905374	None	No
At5g54062	0	F	21956083	21956706	None	No
At5g60945	0	F	24543524	24543862	Transposon	No
At5g60964	0	F	24547812	24548150	Transposon	No
At5g60978	0	T	24559725	24560081	Transposon	Yes

There are 57 members of the CRP3600 subgroup [1]. The Arabidopsis Genome Initiative (AGI) numbers, intron numbers, designations as either gene or pseudogene, and genomic coordinates are taken from Supplementary Figure 2 of the study by Silverstein and coworkers [1]. The methylation status has been determined from our bisulfite data for At4g09545 and from the publicly available methylome data of Lister and coworkers [2]. A ‘Transposon’ methylation pattern indicates relatively dense CG, CHG and CHH methylation in the gene body. ‘Less’ indicates a similar pattern but reduced methylation. Genes or pseudogenes shown in red letters are mentioned in our study. Those shown in blue letters are present in the repeat block of SAT5 monomers close to the centromere of chromosome 5.

[1] Silverstein KA, Moskal WA Jr, Wu HC, Underwood BA, Graham MA, Town CD, VandenBosch KA: **Small cysteine-rich peptides resembling antimicrobial peptides have been under-predicted in plants.** *Plant J* 2007, **51**:262-280.

[2] http://neomorph.salk.edu/epigenome/epigenome.html
