# Supplementary material for: Atypical DNA methylation of genes encoding cysteine-rich peptides in Arabidopsis thaliana
Source: BMC Plant Biol. 2012 Apr 19;12:51. doi: 10.1186/1471-2229-12-51 (PMC3422182; doi:10.1186/1471-2229-12-51)
Supplement: Additional file 4 — Table S3. Primers. [file 1471-2229-12-51-S4.pdf]

Supplementary Table 2. Sequence composition of dispersed SAT5 monomers containing *CRP* genes and pseudogenes

		upstream region		*CRP* coding region		downstream region	
Chr.2	A	139	33,57%	95	25,75%	302	40,54%
(At2g18042)	C	57	13,77%	80	21,68%	73	9,80%
pseudogene	G	51	12,32%	66	17,89%	79	10,60%
	T	167	40,34%	128	34,69%	291	39,06%
	A+T		73,91%		60,44%		79,60%
Chr.3	A	177	35,98%	93	25,83%	154	37,75%
(At3g42565)	C	72	14,63%	80	22,22%	46	11,27%
gene	G	60	12,20%	61	16,94%	49	12,01%
	T	183	37,20%	126	35,00%	159	38,97%
	A+T		73,18%		60,83%		76,72%
Chr.4	A	150	34,64%	95	25,75%	75	35,89%
(At4g09545)	C	60	13,86%	83	22,49%	27	12,92%
gene	G	46	10,62%	63	17,07%	18	8,61%
	T	177	40,88%	128	34,69%	89	42,58%
	A+T		75,52%		60,44%		78,47%
Chr.5	A	66	39,05%	98	27,45%	100	34,17%
(At5g60978)	C	32	18,93%	75	21,01%	41	12,85%
pseudogene	G	14	8,28%	60	16,81%	32	10,03%
	T	57	33,73%	124	34,73%	137	42,95%
	A+T		72,78%		62,18%		77,12%

The numbers of A, C, G and Ts in the indicated regions (upstream region, *CRP* coding region, downstream region) of the SAT5 monomers containing *CRP* genes or pseudogenes (AGI numbers given in column one) are shown together with the percentages for each nucleotide. The percent AT for each region is highlighted in red letters. The two flanking regions are more AT-rich than the *CRP* coding regions.
